# Evolutionary structure of *Plasmodium falciparum* major variant surface antigen genes in South America: Implications for epidemic transmission and surveillance

**DOI:** 10.1002/ece3.3425

**Published:** 2017-10-08

**Authors:** Virginie Rougeron, Kathryn E. Tiedje, Donald S. Chen, Thomas S. Rask, Dionicia Gamboa, Amanda Maestre, Lise Musset, Eric Legrand, Oscar Noya, Erhan Yalcindag, François Renaud, Franck Prugnolle, Karen P. Day

**Affiliations:** ^1^ Department of Microbiology Division of Parasitology New York University School of Medicine New York NY USA; ^2^ MIVEGEC (Laboratoire Maladies Infectieuses et Vecteurs, Ecologie, Génétique, Evolution et Contrôle), UMR CNRS 5290/IRD 224 Université Montpellier 1 Université Montpellier 2 Montpellier France; ^3^ School of BioSciences Bio21 Institute/University of Melbourne Parkville Vic. Australia; ^4^ Instituto de Medicina Tropical Alexander Von Humboldt and Departamento de Ciencias Celulares y Moleculares Facultad de Ciencias y Filosofia Universidad Peruana Cayetano Heredia Lima Peru; ^5^ Grupo Salud y Comunidad Facultad de Medicina Universidad de Antioquía Medellín Colombia; ^6^ Parasitology Unit Institut Pasteur de Guyane Cayenne Cedex French Guiana; ^7^ Unit of Genetics and Genomics on Insect Vectors Institut Pasteur Paris France; ^8^ Centro para Estudios Sobre Malaria Instituto de Altos Estudios en Salud “Dr. Arnoldo Gabaldón” Ministerio del Poder Popular para la Salud and Instituto de Medicina Tropical Universidad Central de Venezuela Caracas Venezuela

**Keywords:** evolutionary structure, *Plasmodium falciparum*, *Plasmodium falciparum* Erythrocyte Membrane Protein 1, population genomics, *var* genes

## Abstract

Strong founder effects resulting from human migration out of Africa have led to geographic variation in single nucleotide polymorphisms (SNPs) and microsatellites (MS) of the malaria parasite, *Plasmodium falciparum*. This is particularly striking in South America where two major founder populations of *P. falciparum* have been identified that are presumed to have arisen from the transatlantic slave trade. Given the importance of the major variant surface antigen of the blood stages of *P. falciparum* as both a virulence factor and target of immunity, we decided to investigate the population genetics of the genes encoding “*Plasmodium falciparum* Erythrocyte Membrane Protein 1” (*Pf* EMP1) among several countries in South America, in order to evaluate the transmission patterns of malaria in this continent. Deep sequencing of the DBLα domain of *var* genes from 128 *P. falciparum* isolates from five locations in South America was completed using a 454 high throughput sequencing protocol. Striking geographic variation in *var* DBLα sequences, similar to that seen for SNPs and MS markers, was observed. Colombia and French Guiana had distinct *var* DBLα sequences, whereas Peru and Venezuela showed an admixture. The importance of such geographic variation to herd immunity and malaria vaccination is discussed.

## 1 INTRODUCTION

There is convincing evidence that *Plasmodium falciparum* originated in Africa and spread to the rest of the world by human migration (Anderson et al., 2000; Duval et al., 2010; Joy et al., 2003; Liu et al., 2010; Prugnolle et al., 2010, 2011; Yalcindag et al., 2012). Strong founder effects, resulting from global migration, have led to *P. falciparum* geographic variation in single nucleotide polymorphisms (SNPs) and microsatellites (MS) with the greatest diversity being observed within Africa. This spatial variation is particularly striking in South America where two main genetic clusters, previously shown through SNPs and MS variation, suggest independent introductions of *P. falciparum* from Africa through the transatlantic slave trade about 500 years ago (Yalcindag et al., 2012). Yalcindag and colleagues provided evidence for structuring of the parasite population into a northwestern cluster (Colombia) and a southeastern cluster (French Guiana/Brazil/Bolivia; Yalcindag et al. 2012). This structure is believed to have originated through distinct human population movements related to the subdivision of the continent into the Portuguese and Spanish empires and is maintained today through the admixture of populations (Venezuelan and Peruvian) between these two clusters. The genetic markers used in the study of *P. falciparum* population structure in South America by Yalcindag et al. were putatively neutral allowing for inference of population history as well as demographic events (Yalcindag et al., 2012). They, however, do not define characteristics of parasite fitness (Kirk & Freeland, 2011). By comparison, both drug resistance markers and variant antigen encoding loci provide information about parasite behavior in relation to drug and/or immune selection forces. These biomarkers are the key to pathogen diagnostic surveillance as they allow for the prediction of epidemics with different behaviors over space and time.

We decided to investigate the evolution of *var* genes, encoded by the major blood stage variant surface antigen, “*Plasmodium falciparum* Erythrocyte Membrane Protein 1” (*Pf*EMP1), among several countries in South America to evaluate the population structure of these genes at the scale of a continent, and thus describe the transmission patterns of malaria. The *P. falciparum* genome is composed of up to 60 *var* genes with each representing a different antigenic form. To achieve clonal antigenic variation within the host, *Pf*EMP1 is expressed sequentially in a mutually exclusive manner (Dzikowski, Frank, & Deitsch, 2006; Scherf, Lopez‐Rubio, & Riviere, 2008; Voss et al., 2006). This remarkable biological feature enables *P. falciparum* to evade the human immune response and establish chronic infections linked to specific cellular interactions. Indeed, *Pf*EMP1 also binds to host endothelial tissues with different variants exhibiting specific adherence characteristics for tissues, which in turn are associated with distinct disease manifestations (Avril et al., 2012; Claessens et al., 2012; Kraemer & Smith, 2003). *Pf*EMP1 is thereby considered a virulence factor. The *var* multigene family is highly diverse among parasite genomes in natural parasite populations as well as in clinical cases (Mugasa et al., 2012; Rorick et al., 2013; Sulistyaningsih et al., 2013; Warimwe et al., 2013). The *var* gene family contains several semi‐conserved domains with specific structural characteristics called Duffy Binding‐Like (DBL) domains. Among the different DBL domains characterized, DBLα is the most conserved and is involved in the adherence of infected red blood cells (RBCs) to uninfected RBCs (Chen et al., 1998; Vogt et al., 2003). Bioinformatic analyses have shown that *var* gene diversity is of ancient origin and maintained by balancing selection, and that through the process of shuffling homology blocks during recombination *var* genes are able to diversify (Rask et al., 2010; Zilversmit et al., 2013). Moreover, it has been demonstrated that *var* gene repertoire diversity within and between parasite genomes is also generated by meiosis during sexual recombination (Chen et al., 2011; Zilversmit et al., 2013) and by mitotic recombination during asexual division (Bopp et al., 2013; Claessens et al., 2014; Duffy et al., 2009). The high levels of mitotic recombination observed within cloned lines have led to a view that *var* genes may not be a stable marker for molecular surveillance. However, population genetic studies from malaria endemic regions like Africa, Papua New Guinea, and South America have demonstrated that sequencing the highly conserved DBLα domain of *var* genes is an effective approach to both characterize and monitor *P. falciparum* diversity spatially and longitudinally (Barry et al., 2007; Chen et al., 2011; Day et al., 2017; Scherf et al., 2008; Tessema et al., 2015).

To date, limited population sampling of *var* genes from Venezuela and Brazil in South America has revealed restricted diversity both locally and regionally, as compared to African populations (Albrecht et al., 2006, 2010; Chen et al., 2011; Tami et al., 2003). Structuring of *var* genes across local populations on the South American continent, however, has not been established. In this study, using next‐generation 454 sequencing, we have successfully genotyped the *var* DBLα domains of 128 *P. falciparum* clinical field isolates collected from four countries in South America between 2002 and 2008 and for which SNP and MS data were available for comparison (Yalcindag et al., 2012). Population genetic analysis using *var* genes has allowed us to address the following questions: (i) What are the limits of *var* gene diversity in these South American populations? (ii) Does population structuring at *var* loci exist between/among the South American populations?, and (iii) Are *var* gene population genetics a reflection of geographic population structure on the South American continent when compared to SNPs and MS markers? The answers to these molecular epidemiological questions would allow us to predict the potential for epidemic transmission of *P. falciparum* clones not previously observed across South America.

## 2 MATERIALS AND METHODS

### 2.1 Ethical statement

Ethical clearance was obtained from the local ethics committees in each country sampled. The informed consent procedure for the study consisted of a presentation of the aims of the study to the community followed by invitation of individuals (or their parents/guardians) for enrollment. At the time of sample collection, the purpose and design of the study was explained to each individual and verbal informed consent was collected by a minimum of two people. The verbal consent process was consistent with the ethical expectations for each country at the time of enrollment, and the ethics committees approved these procedures.

All the samples collected from French Guiana and analyzed in this study were from blood collections that were required as standard medical care for any patient presenting with a fever on admission to the hospital. According to French legislation (Article L.1211‐2 and related, French Public Health Code), biobanking and the secondary use of remaining human clinical samples for scientific purposes is possible if the corresponding patient is informed and has not objected to such use. This requirement was fulfilled for the present study; each patient was informed via the hospital brochure entitled “Information for Patients,” and no immediate or delayed patient opposition was reported to the Malaria NRC by the clinicians.

For samples collected in Colombia, each patient (or their parents/guardians) gave informed written consent. Ethical clearance was granted by the Ethics Committee of the Centro de Investigaciones Médicas, Facultad de Medicina, Universidad de Antioquia (Medellín, Colombia).

Concerning the Peruvian samples, the study protocol was approved by both the Ethical Review Committee of the Universidad Peruana Cayetano Heredia and the Institute of Tropical Medicine, Antwerp, Belgium. The research was performed in accordance with the ethical standards of the Peruvian Ministry of Health. The trial has been registered as an International Standard Randomised Controlled Trial, number NCT00373607 at http://www.clinicaltrials.gov.

For samples collected in Venezuela, each patient gave written informed consent and ethical clearance was obtained from the Comité Ético Científico del Instituto de Medicina Tropical de la Universidad Central de Venezuela.

### 2.2 Study samples

**Figure 1:**
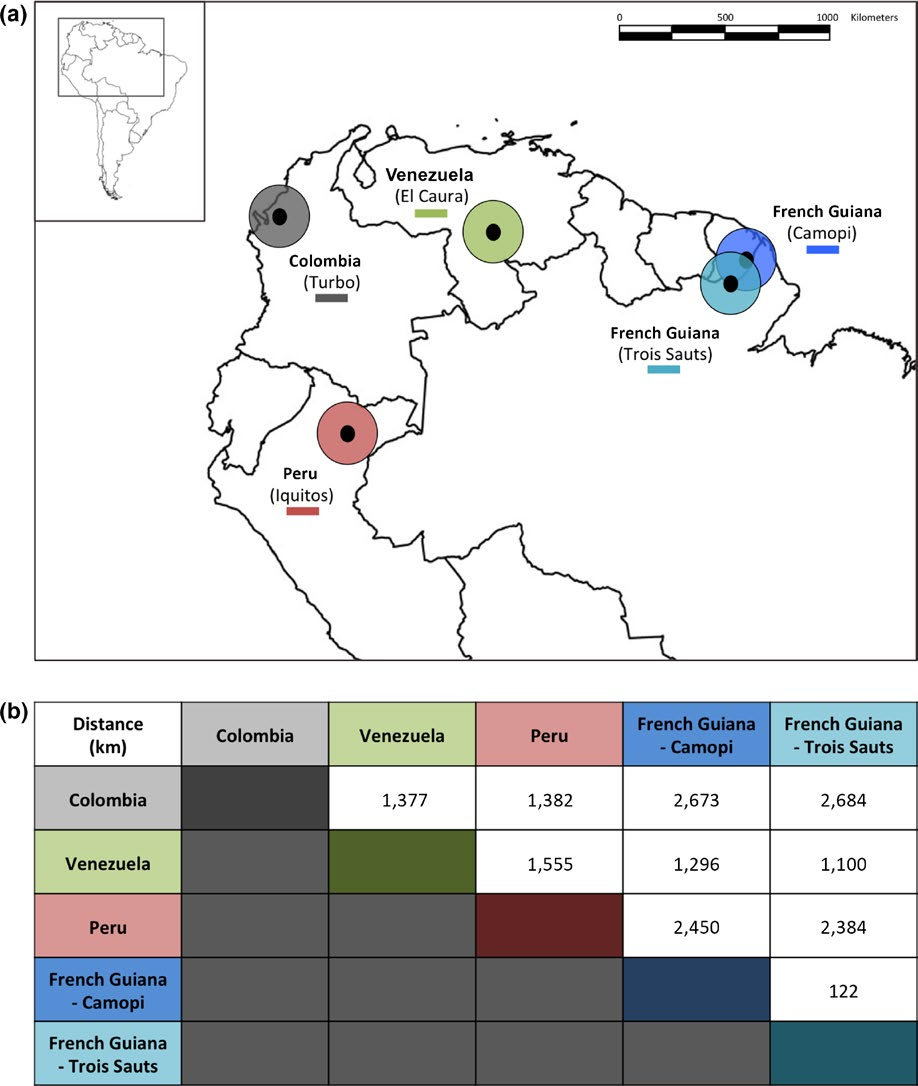
Map of South America showing the five study site locations/populations. (a) Each study site is denoted with a colored circle: Colombia (grey), Venezuela (green), Peru (red), French Guiana–Camopi (blue), and French Guiana–Trois Sauts (turquoise). The locations of these sites within the South American continent are presented in the insert map (upper left). (b) Calculated distance between each South American population

The 128 *P. falciparum* isolates typed in this study (Table 1) originated from the collections previously described in Yalcindag et al. (2012). These isolates were collected between 2002 and 2008 from individuals presenting with clinical malaria from five study site locations in four countries from South America: Peru (Iquitos), Venezuela (El Caura), Colombia (Turbo), and French Guiana (Camopi and Trois Sauts) (Figure 1a). Each of the five populations was represented and between 10 and 41 isolates were included in the study depending on geographic location. *P. falciparum‐*infected blood samples were collected by either venous puncture (~500 μl) or by finger prick (~50 μl) after obtaining informed consent.

**Table 1:** Population characteristics, *var* DBLα type sampling and estimated *var* DBLα type richness for the South American populations surveyed

Country (Population)	Dates of collection	Number of isolates	Median DBLα repertoire size (min–max)	Number of non‐redundant DBLα sequences	Observed number of unique DBLα types	Chao1 richness estimates (95% CI)
Colombia (Turbo)	2002–2004	21	40 (19–43)	807	112	117 (113–133)
Venezuela (El Caura)	2003–2007	10	36.5 (28–43)	352	176	257 (223–314)
Peru (Iquitos)	2003–2004	21	36 (19–42)	702	157	207 (179–268)
French Guiana (Camopi)	2006–2008	41	45 (13‐92)	2,048	229	280 (250–351)
French Guiana (Trois Sauts)	2006–2008	35	50 (36–82)	1,790	210	223 (215–245)
All Populations	///	128	42 (13–92)	5,699	458	536 (502–596)

### 2.3 Genotyping

Single nucleotide polymorphisms (SNPs) and microsatellite (MS) genotyping were previously performed in the study of Yalcindag et al. (2012). These data were used in the present analyses for comparison.

### 2.4 PCR amplification for *var* DBLα typing

DNA from blood samples was extracted using the DNeasy Blood and Tissue Kit (Qiagen, France) according to the manufacturer's recommendations and eluted in 100 μl of elution buffer per 200 μl of whole blood or per dried filter blot. The conserved *var* DBLα domain has previously been used as a marker for *var* gene diversity in other global studies (Barry et al., 2007; Chen et al., 2011; Day et al., 2017; Tessema et al., 2015). The *P. falciparum var* DBLα domain was amplified from genomic DNA using fusion primers for multiplexed 454 Titanium sequencing. We coupled template‐specific degenerated primer sequences to blocks D and H (Bull et al., 2007): DBLα AF, 5′‐GCACGMAGTTTYGC‐3′, and DBLα BR, 5′‐GCCCATTCSTCGAACCA‐ 3′. Specifically, forward and reverse primers were designed by adding a GS FLX Titanium Primer sequence 10‐bp multiplex identifier (MID) tags published by Roche (Roche 454 Sequencing Technical Bulletin No. 013‐2009; 454 Sequencing Technical Bulletin No. 005‐2009). These MIDs have been engineered to avoid miss assignment of reads and are tolerant to several errors. A full list of the primer sequences utilized in this study can be found in Tables S1 and S2. This method of MID tagging isolates has been previously described and validated with *P. falciparum* reference strains (3D7, Dd2, HB3) (Rask et al., 2016). All PCR reactions were carried out in a total volume of 40 μl consisting of 0.5× buffer, 1.25 mmol/L MgCl_2_, 0.07 mmol/L dNTP mix, 0.375 μmol/L of each primer (forward and reverse), 0.075 units of GoTaq DNA Polymerase (Promega), 2 μl of purified genomic DNA template, and 27 μl of water. Each isolate was amplified using forward and reverse primers containing the same MID tag combination. Amplifications were carried out on an Eppendorf EP Gradient Mastercycler using the following reaction conditions: 95°C for 2 min, followed by 30 cycles of 95°C for 40 s, 49°C for 1 min 30 s, 65°C for 1 min 30 s, and a final extension step of 65°C for 10 min. PCR amplification was confirmed visually by nucleic acid staining (EZ VISION^™^ DNA Dye, Amresco) followed by gel electrophoresis (1.5% agarose in 0.5× TBE buffer) demonstrating a band of the appropriate size (~550–700 bp). Positive controls (laboratory genomic *P. falciparum* DNA) and negative control (no template) were included for quality assurance.

The PCR products were purified using the SPRI method (solid‐phase reversible immobilization) (Agencourt, AMPure XP), and PCR amplicon concentrations were measured using the Quant‐iT PicoGreen dsDNA Kit per the manufacturer's instructions (Invitrogen). Known concentrations of control DNA were prepared as directed by the Roche Technical Bulletin (454 Sequencing Technical Bulletin No. 005‐2009). We assayed fluorescence intensity using a PerkinElmer VICTOR X3 multilabel plate reader, with fluorescein excitation wavelength of ~480 nm and emission of ~520 nm wavelength. We prepared four PCR amplicon library pools, each containing equimolar amounts of up to 60 PCR amplicons all with unique MID tags. These four pools were sequenced in the forward and reverse directions on segregated regions of one full 454 plate using GS FLX Titanium chemistry (Roche). This 454 high throughput sequencing approach provides average read lengths of 400 bp, therefore lending itself to the assembly of the individual *var* DBLα amplicons of 550‐ to 700‐bp lengths using the forward and reverse sequence reads from each direction. Sequencing was performed by Seqwright Genomics (Houston, TX, USA).

### 2.5 *Var* DBLα sequence analysis

A custom pipeline was developed to demultiplex, denoise, and remove PCR and sequencing artefacts from the DBLα domain reads. The first part of the pipeline is available as the Multipass web server: http://www.cbs.dtu.dk/services/MultiPass-1.0, and the following cleaning steps described below are implemented in a python script available here: https://github.com/454data/postprocess. The sff‐files obtained from each region on the 454‐plate were divided into smaller isolate‐specific sff‐files by identification of reads with exact matching MID sequences in both ends using BioPython v1.57 (Cock et al., 2009). Ambiguous primer sites were then identified (exact match) and trimmed off the flowgrams, reverse reads were reverse complemented, and a dat‐file (AmpliconNoise format) with the resulting flowgrams was created for each isolate, using BioPython v1.57 (Cock et al., 2009). By combining the forward and reverse reads, this method takes advantage of bidirectional amplicon sequencing, since the forward reads will have highest quality in the 5′‐end of the target sequence, and the reverse reads will improve the 3′‐end quality.

Flowgram clustering was performed using PyroDist, FCluster, and PyroNoiseM from the AmpliconNoise package v1.25 (Quince et al., 2011). The flowgram clusters produced by AmpliconNoise were base called using Multipass to obtain the most likely DBLα sequences given a full length open reading frame (FRF) probability of *p*(FRF) = .9979 as described in Rask et al. (Rask et al., 2016); however, an alternate flow calibration was used. Control isolate flow value distributions for the longest homopolymers in this 454 run differed slightly from the previously described normal distributions (Balzer et al., 2010). This phenomenon was also observed for another 454 run performed at the same sequencing facility in connection with another study. Maximum‐likelihood fitting showed that flow values from homopolymers of 1–5 nucleotides were optimally described by normal distributions, whereas homopolymers of length >5 were most accurately modeled by log‐normal distributions (Fig. S1). This finding emphasizes the importance of including control samples in each sequencing run for calibration purposes. Parameters for the log‐normal distributions fitted to transformed flow values *s*_*t*_ can be found in Fig. S1. The transformation consists of a negation and a shift along the abscissa:

$$s_{t}=h+2-s$$

where *h* is the length of the homopolymer that gave rise to the flow value *s*. So the log‐normal probability density function is:

$$\text{PDF}\left(s|h,\mu ,\sigma \right)=\frac{1}{\left(h+2-s\right)\sigma \sqrt{2\pi }}e^{\frac{{\left(\ln(h+2-s)-\mu \right)}^{2}}{2\sigma^{2}}}$$

Parameter extrapolation was performed to obtain expected flow distributions for homopolymer lengths that were rare in the control isolates (Fig. S1).

The nucleotide sequences generated by Multipass were clustered by 96% identity using Usearch v5.2.32 (Edgar, 2010; Edgar & Flyvbjerg, 2014) with seeds (cluster member with support from highest number of reads after dereplication) as output. Chimeras were removed using Uchime implemented in Usearch v5.2.32 (Edgar, 2010; Edgar et al., 2011), first in de novo mode where chimera detection is based on read abundance, all parents are expected to be present in the sequence set, and candidate parents must be at least 2× more abundant than the chimera candidate sequence. Subsequently, database mode was applied, where sequences are searched against self and chimeras are found irrespective of the abundance of the parents. To increase overall sequence quality, a minimal coverage threshold of three reads per sequence type was applied to remove the least supported sequences. Next, we screened for and removed nontarget amplified human sequences by local alignment search against the BLAST human genomic databases (ftp://ftp.ncbi.nlm.nih.gov/blast/db/) using the blastn feature of BLAST+ 2.2.25 (NCBI), with expectation value criteria of 1*e*−50 (90 sequences removed). Sequences were also BLASTed against the remaining (non‐DBLα) 3D7 genome and searched using a DBLβ HMM with HMMer v3.1 (http://hmmer.org); however, no hits were found. After the human and nontarget *P. falciparum* check, a small number of sequences remained that had no similarity to a DBLα tag HMM and these were removed. The pipeline was validated and optimized on experimental sequence data generated on the laboratory clones (3D7, Dd2, HB3) for which published genome sequences are available. More than 90% of the sequences obtained from the control samples had no errors when compared to the known references, and the deviating sequences had only one to three errors. To define distinct DBLα types shared within and between populations, we clustered nonredundant sequences from all isolates from the South American populations by average linkage using a sequence identity threshold of 96%. This 96% cut off was chosen to define unique DBLα types as it has been previously shown to be robust at defining DBLα types with identical sequences (excluding minor sequence errors) (Day et al., 2017).

### 2.6 Diversity analysis

#### 2.6.1 Richness estimates

Using EstimateS v9.1 (Colwell, 2013), the diversity of DBLα types within and among all sites in South America was calculated by estimating the total number of DBLα types and the proportion of DBLα types shared between isolates. For each South American population, nonparametric statistical estimates of richness, Chao1, and 95% confidence intervals were calculated. The Chao1 statistic estimates the total number of types in a population using frequency data on types seen once only and types seen twice only (Chao, 1984). In the setting of equal probability of distribution and sampling of types, the Chao1 estimator yields a point estimate of richness. These estimators cannot predict a probable maximum richness.

#### 2.6.2 Cumulative diversity curves

The cumulative diversity curves, analogous to species accumulation, were generated using EstimateS v9.1 (Colwell, 2013) to estimate DBLα richness by sampling all DBLα types within each South American population and among all populations without replacement. The cumulative diversity curve plots the number of unique DBLα types as a function of the number of DBLα sequences sampled. The curves were plotted in Microsoft Excel.

#### 2.6.3 Similarity indices

Ecological indicators of similarity were calculated to quantify the relatedness between the *var* gene repertoires identified from two isolates and among the South American populations using the DBLα domain.

#### 2.6.4 Pairwise type sharing

Pairwise type sharing (PTS), analogous to Sørensen's Index (quotient of similarity, or QS), is a useful statistic to analyze diversity and determine the proportion of DBLα types shared between isolate repertoires and among the South American populations (Barry et al., 2007; Chao, Chazdon, & Shen, 2005). If isolate A has a repertoire of *n*_A_ unique DBLα types, isolate B has a repertoire of *n*_B_ unique DBLα types, and a total *n*_AB_ DBLα types are shared by the isolates A and B; PTS (or QS) is defined as: PTS_AB_ *= *2*n*_AB_/*n*_A_ *+ n*_B_. PTS values range from 0 to 1, where a PTS score of 0 signifies no DBLα type sharing and 1 signifies complete sharing of all DBLα types.

Additionally, the PTS statistic was used to generate a distance matrix with genetic distance being defined as pairwise type distance (PTD). PTD was calculated as follows: PTD_AB_ *= *1 − PTS_AB_. PTD values range from 0 to 1, where a PTS score of 0 signifies that two isolate repertoires or populations are genetically identical, while 1 signifies that they are genetically distinct. The relationships were visualized using a neighbor joining tree constructed using Clearcut v1.0.9 (Barry et al., 2007; Sheneman, Evans, & Foster, 2006). A tree was also constructed using Jaccard distances (Jaccard, 1912); however, the tree based on PTD captured the geographic structure more clearly as it accounted for (i) the large number of DBLα types in the population and (ii) was weighted based on the abundance of DBLα types (i.e., DBLα types shared between isolates have more weight).

#### 2.6.5 Chao–Sørensen's Index

Chao–Sørensen's Index provides another estimate of similarity among the South American populations. This index adjusts for the abundance of each DBLα type (not just presence or absence of the DBLα type) in the population, as well as adjusting for the effect of unseen shared DBLα types in conditions of under sampling (Chao et al., 2006). These calculations were performed using EstimateS v9.1 (Colwell, 2013).

Similar to PTS (or QS), the Chao–Sørensen's Index was also used to determine genetic distance and the level of genetic differentiation between the South American isolates/populations using DBLα types. This measure was defined as Chao–Sørensen's Quotient of Distance (Chao–Sørensen's QD) and was calculated as: *Chao–Sørensen's* QD_AB_ *= *1 − (Chao–Sørensen's Index)_AB_.

### 2.7 Genetic diversity and genetic differentiation

The distribution of genetic diversity of DBLα types among the South American populations was investigated using the same methods and tools previously described in Yalcindag et al. (2012). For these analyses, each DBLα type was considered a locus and each isolates’ multilocus genotype was the sum of the presence (coded as 1) or absence (coded as 2) of each DBLα type. For each isolate, all unique DBLα types identified were included.

#### 2.7.1 Genetic differentiation

Pairwise Weir and Cockerham's *F*_ST_ estimates (Weir & Cockerham, 1984) between the South American population were computed for the SNPs, MS, and DBLα types using the FSTAT V.3.7 software (updated from (Goudet, 2001)). To explain patterns of isolation by distance (IBD) among the South American populations, we evaluated the Pearson's correlation coefficient (*r*) between the various indices of genetic distance (*F*_ST_, PTD, Chao–Sørensen's QD) for SNPs, MS, and DBLα types, and geographical distance between each South American population pair. Pairwise geographic distances were computed using MapInfo (Pitney Bowes Business Insight, Troy, NY). The significance of the relationship was assessed with a Mantel test using 10,000 permutations. In addition, the genetic distance (*F*_ST_) of the SNP markers among the South American populations was compared with the other indices of genetic distances for the MS (*F*_ST_) and the DBLα types (*F*_ST_, PTD, Chao–Sørensen's QD) for the South American populations. All Pearson's correlation coefficients (*r*) were calculated in Microsoft Excel.

#### 2.7.2 Principal component analyses

Principal component analyses (PCA) were performed on the matrix of binary allele profiles using the R‐package “Adegenet” (Jombart, 2008). These analyses were completed to obtain further understanding on the genetic structure of the South American isolates and populations.

#### 2.7.3 STRUCTURE analyses

We used the Bayesian clustering method implemented in STRUCTURE v.2.1 (Pritchard, Stephens, & Donnelly, 2000) to identify population structure. We ran models allowing for admixture, with the number of clusters or populations (*K*), ranging from *K* = 1 to the number of South American populations included (*K* = 5) ((Raymond & Rousset, 1995; Anderson et al., 2000), depending on the dataset). All simulations used 100,000 Markov chain Monte Carlo (MCMC) generations in the burn‐in phase and 100,000 generations in the data collection phase. Ten independent runs were performed for each specified *K* to verify convergence in the estimates of posterior probabilities. The optimal number of clusters was estimated using the method proposed in Evanno, Regnaut, and Goudet (2005) (Evanno et al., 2005).

## 3 RESULTS

### 3.1 Summary of sequencing results

The *var* DBLα domains of the 128 clinical field isolates collected previously in South America (Yalcindag et al., 2012) were successfully sequenced using the next‐generation 454 sequencing (Roche) approach. After the application of the custom pipeline for DBLα sequence analysis described above, 169,862 sequence reads remained for the South American populations. The mean read length was 400 bp. Following the application of quality control measures, the 128 isolates collected from South America had DBLα sequence reads available for further analyses with a mean coverage of 1,327 reads per isolate. The distribution of reads obtained per isolate, by population, is presented in Fig. S2.

### 3.2 Assembly of reads into *var* DBLα sequences

Within each isolate, sequences were clustered into nonredundant DBLα sequences using the flowgram clustering method (Section 2). The method resulted in 5,699 DBLα sequences among the 128 South American isolates with 29.8 sequence reads per DBLα sequence. Among the South American populations, between 352 and 2,048, nonredundant DBLα sequences were identified (Table 1) and represent the dataset on which the analyses were performed.

### 3.3 Definition of *var* DBLα types, frequency distribution, and richness estimates

To determine the number of unique DBLα types shared between isolates/populations, we clustered the nonredundant DBLα sequences from all isolates at 96% pairwise identity. This resulted in 458 unique DBLα types (median = 176, range = 112–229 DBLα types per population) among the 128 isolates from the five South American populations (Table 1).

**Figure 2:**
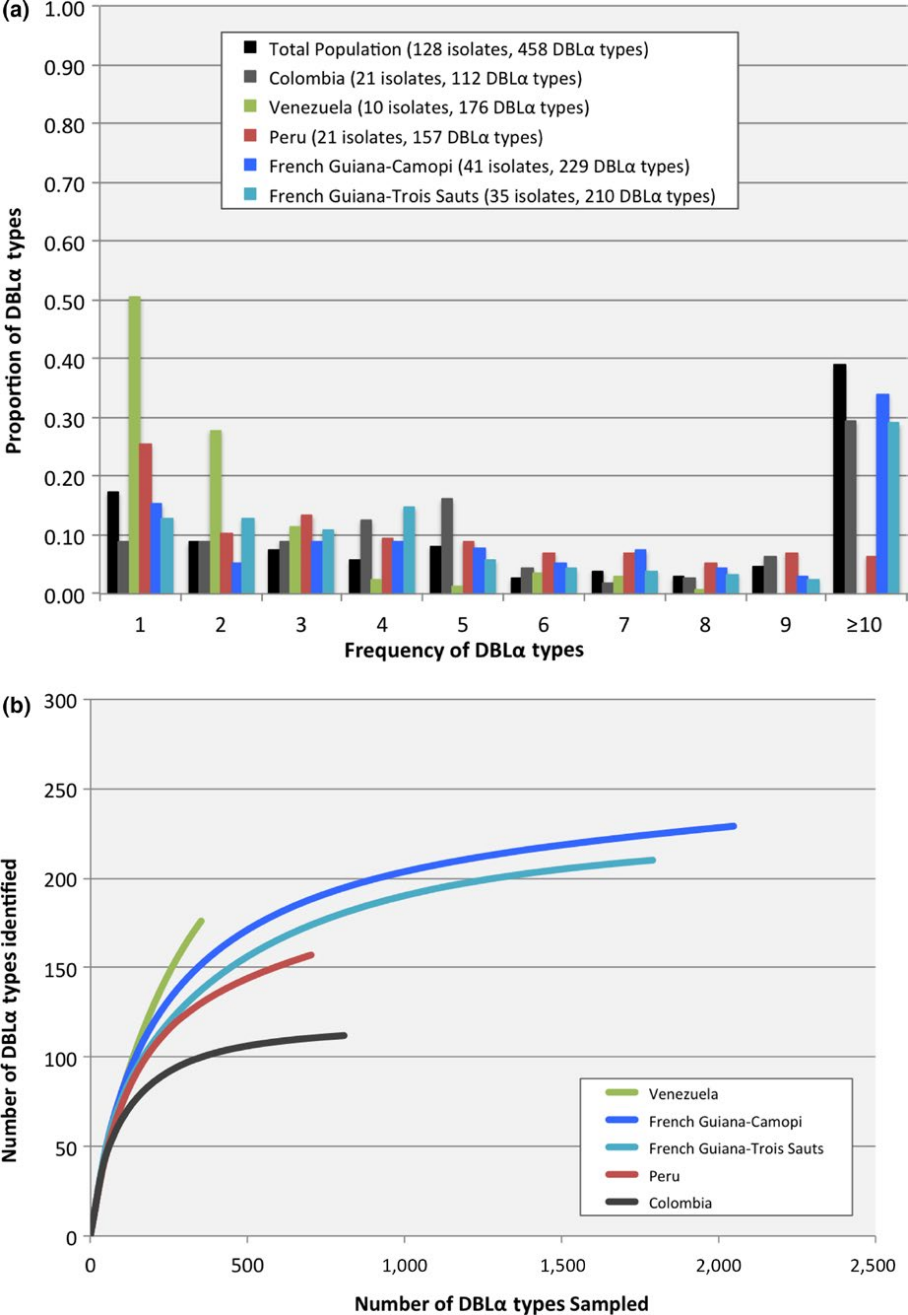
Frequency distribution and diversity of *var* DBLα types in the population. (a) For the South American isolates, clustering of the 5,699 DBLα sequences resulted in 458 unique *var* DBLα types. The figure provides the proportion of *var* DBLα types that appeared one to ten or more times within and among all the South American populations. (b) Cumulative diversity curves for the *var* DBLα types sampled in each South American population. The Venezuelan curve (green) does not show evidence of leveling off, in contrast to those for Colombia (grey), Peru (red), French Guiana‐Camopi (blue) and French Guiana–Trois Sauts (turquoise). The up‐sloping curve for Venezuela suggests that more DBLα types will be found with further sampling of this population

Within and among the South American populations, the distribution of the DBLα types showed similar patterns of abundance, except for Venezuela, which had the smallest number of isolates (*N* = 10) available for comparison (Figures 2a and S3). Among the South American populations surveyed, the majority of DBLα types reoccurred and were observed in >1 isolate (379, 82.6%), with 178 DBLα types (38.9%) being considered abundant as they were seen in ≥10 isolates (Figure 2a). Within each population, the proportion of DBLα types that reoccurred (>1 isolate) ranged from 49.7% in Venezuela to 91.1% in Colombia (Figure 2a and S3). In comparison with Venezuela where more than half of the of DBLα types were rare (only seen once among the isolates sampled), the majority of the DBLα types observed in Colombia, Peru, and French Guiana (Camopi and Trois Sauts) reoccurred and were seen in more than one isolate (range = 74.7%–91.1%) (Figure 2a and S3). In Venezuela, the absence of abundant DBLα types (≥10 isolates) in the population is perhaps the result of significantly under sampling the local *P. falciparum* population in comparison with the other South American countries surveyed, leading to an alternate frequency distribution of DBLα types.

Using the richness estimator Chao1, we estimated the number of DBLα types within each population and they ranged from 117 to 280 (Table 1); for the combined South American populations, it was estimated that there would be 536 DBLα types in South America (Table 1). We measured depth of DBLα type sampling in each population with a rarefaction curve depicting the rate at which new DBLα types were identified with the collection of unique DBLα sequences from each isolate. Deep sampling of the DBLα types was achieved within the South American populations surveyed as evidenced by the flattening of the rarefaction curves, with the exception of Venezuela, which did not reach saturation in sampling evidenced by its failure to level off (Figure 2b). This failure to level off further indicates that the Venezuelan population was undersampled, and that additional *P. falciparum* isolates are necessary for more thorough within and among population comparisons.

**Figure 3:**
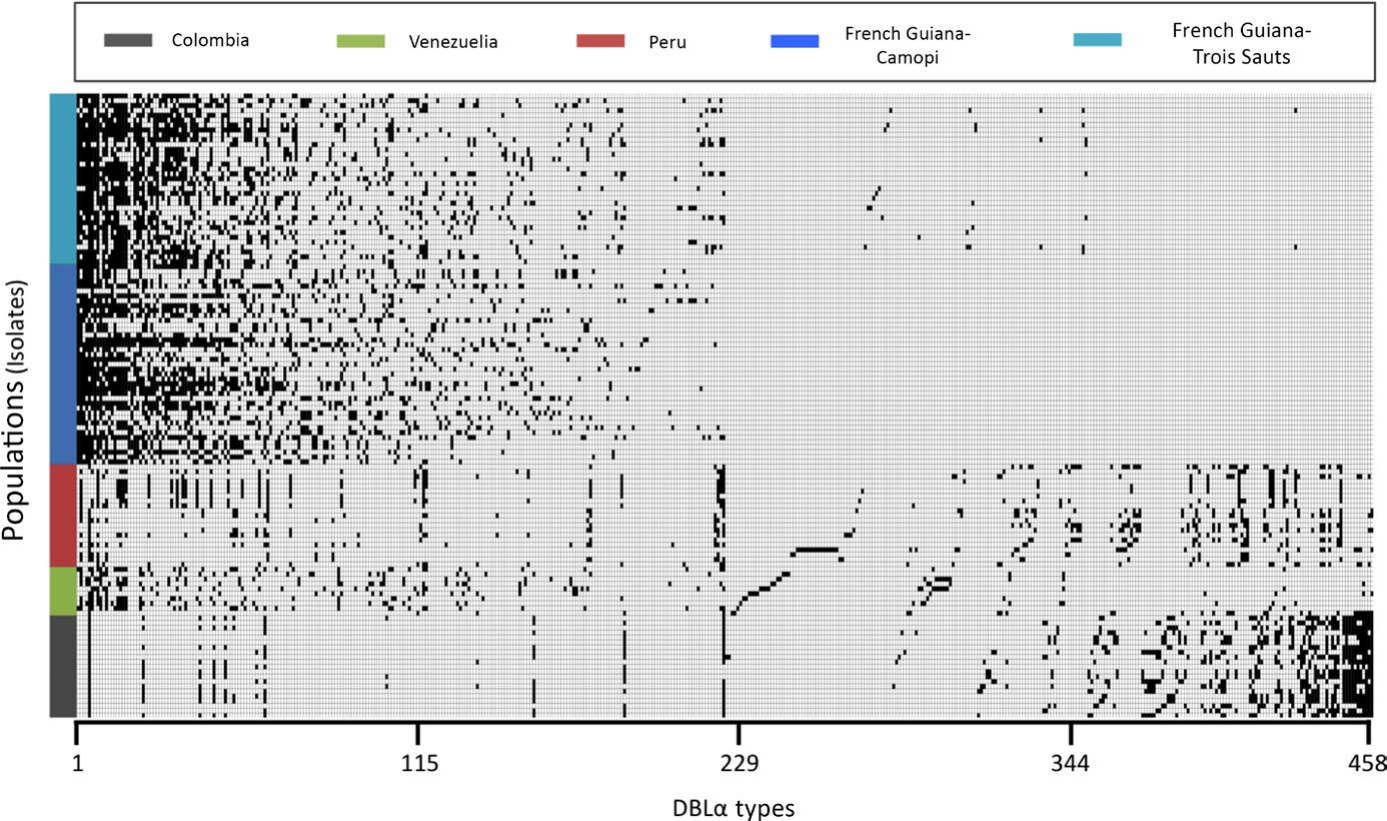
Distribution and organization of the *var* DBLα types among *Plasmodium falciparum* isolates from the South American populations surveyed. A presence–absence matrix for the 458 unique *var* DBLα types (indicated on the *x*‐axis) in each of the 128 South American isolates (indicated on the *y*‐axis), where the black boxes represent the presence of a *var* DBLα type in an isolate. The *var* DBLα types are ordered from left to right (on the *x*‐axis) by decreasing frequency based on the French Guiana–Camopi isolates (largest number of unique DBLα types), to permit comparisons of prevalence and membership for each of the 458 DBLα types across all five populations surveyed

To understand patterns of DBLα type co‐occurrence among isolates and within populations, each of the 458 unique DBLα types was plotted against all isolates surveyed in South America (Figure 3). When the presence/absence of the DBLα types was examined, it was evident that there was: (i) sharing of DBLα types among isolate repertoires, (ii) reoccurrence of DBLα types (>1 isolate) within and among the South American populations, and (iii) conservation of abundant DBLα types (≥10 isolates) within and among populations signifying underlying geographic population structure in South America. The isolates from French Guiana grouped together and were distinct from those sampled in Colombia, with the Venezuelan and Peruvian isolates being distributed between each of these two separate geographic clusters (Figure 3).

### 3.4 Analysis of isolate *var* DBLα repertoires

**Figure 4:**
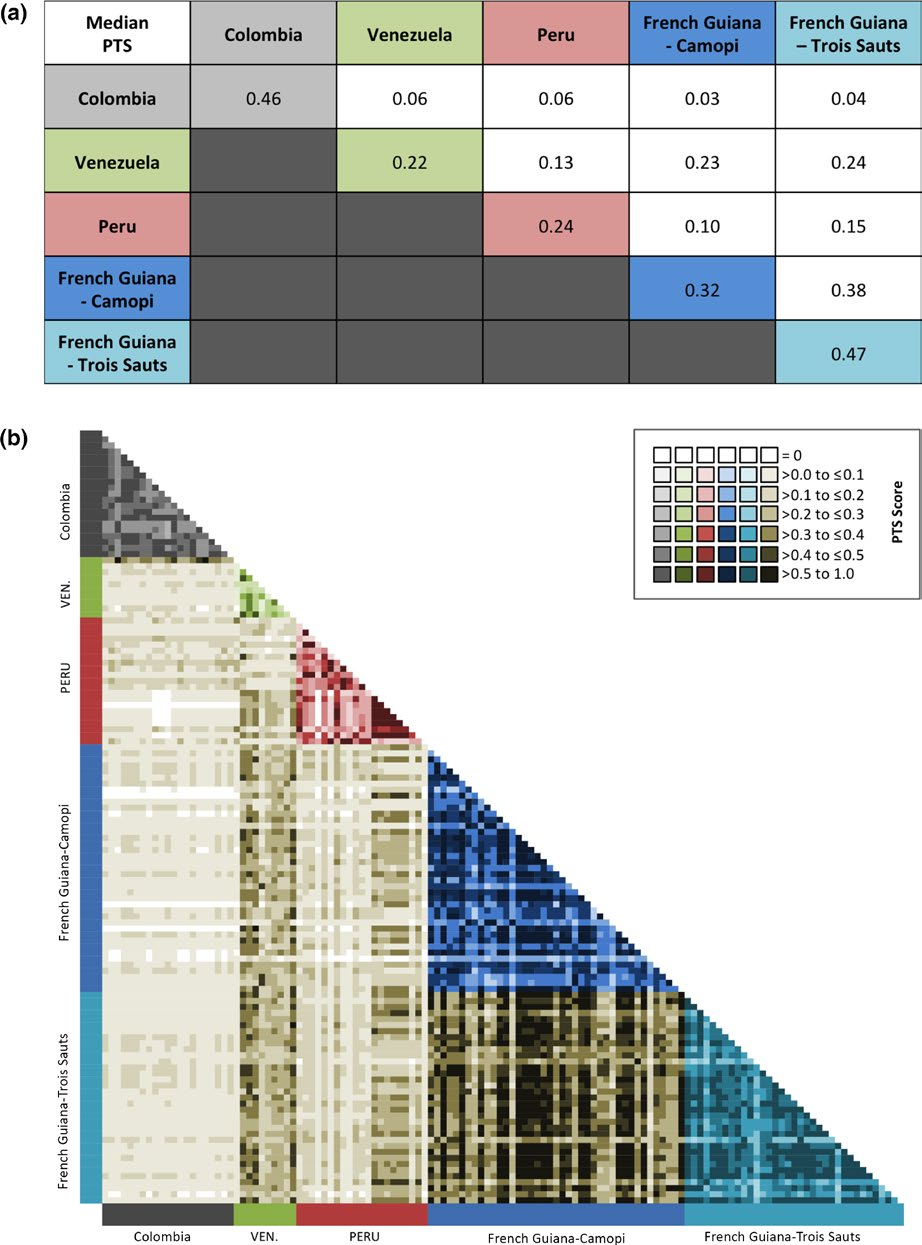
Pairwise comparisons of the *var* DBLα repertoire overlap. (a) The median pairwise type sharing (PTS) scores calculated for all possible pairwise comparisons between the isolates within and among the South America populations. (b) Heat map representation of the PTS of the *var* DBLα types among isolates within and among the South American populations. Different color shading was used to denote the PTS values within and among the populations (Colombia (grey), Venezuela (green), Peru (red), French Guiana–Camopi (blue), French Guiana–Trois Sauts (turquoise), and between all sites (taupe)). *Note:* As indicated in the color key provided (upper right corner), the darker the color shading the greater the *var* DBLα repertoire overlap is between the two isolates being compared, while no shading indicates a PTS score of zero (i.e., no sharing)

At the isolate level, the median repertoire size (number of unique DBLα types per isolate) was 42 (range = 13–92); however, the majority of isolates (*N* = 125, 97.7%) had repertoires composed of ≥20 DBLα types (Fig. S4); that is, they were sufficiently well sampled to permit comparison of DBLα types between isolate repertoires. To quantify DBLα repertoire overlap between isolates both within and among the South American populations, PTS was calculated as a similarity index (see Section 2). The median PTS scores within each of the South American populations ranged from a maximum of 0.46 between the isolate repertoires in Colombia to a minimum of 0.22 in Venezuela (Figure 4a). By using PTS, we observed similar spatial patterns of geographic differentiation among the South American populations. The median PTS scores between the DBLα repertoires ranged from a maximum of 0.38 between the French Guiana isolates of Camopi and Trois Sauts, to a minimum of 0.03 between the isolates in Colombia and French Guiana (Camopi) (Figure 4a). The isolates from French Guiana clustered together (i.e., higher median PTS scores, darker shading on the PTS heat map) and were distinct from those isolates sampled in Colombia (i.e., lower median PTS scores, lighter shading on the PTS heat map) (Figure 4b). The Peruvian and Venezuelan isolates’ median PTS scores were distributed between these two geographic clusters, as they showed transitional DBLα repertoire overlap (i.e., moderate shading on the PTS heat map) with both the French Guiana and Colombian isolates (Figure 4b).

### 3.5 Geographic structuring of the South American *P. falciparum* isolates

The study of the structural genetic organization of the South American populations, based on different analytic strategies, showed distinct features that were consistent with those obtained from SNPs and MS (Yalcindag et al., 2012): (i) The isolates from the French Guiana populations formed a distinct cluster, (ii) the Colombian isolates were closely related and well separated from the other populations included in this analysis, and (iii) the Peruvian and Venezuelan populations were situated in an intermediate position between Colombia and French Guiana. Notably, the Venezuelan isolates seemed to be genetically closer to the isolates from French Guiana than to those from Peru. However, this observation should be taken with caution since the DBLα types sequenced in Venezuela were under sampled compared to the other South American locations included in this study (Figure 2b).

To assess the structuring of DBLα types on a geographical scale in South America, we investigated the number of shared DBLα types between the populations by calculating the PTS score for each population pair (Table 2). Based on the PTS scores, there was geographic variability in the sharing of DBLα types between the South America populations. As evidenced by the low PTS scores, the Colombian parasite population was distinct from the population surveyed in French Guiana (i.e., 0.08 when comparing Colombia to either Camopi or Trois Sauts from French Guiana) (Table 2). In contrast, Peru and Venezuela showed intermediate DBLα type sharing with each other and with the Colombian and the French Guiana clusters (i.e., PTS scores varied between 0.24 and 0.52) (Table 2). Meanwhile, the populations in French Guiana appeared nearly indistinguishable with 87% of their DBLα types being shared (Table 2).

**Table 2:** Sharing of *var* DBLα types among the South American populations. The number of unique *var* DBLα types for each South American population as well as the number of shared *var* DBLα types between each of the South American population pairs. The pairwise type sharing (PTS) scores (representing the proportion of *var* DBLα types shared between two populations) was calculated for all possible pairwise comparisons. A score of 0 represents no sharing of *var* DBLα types between the populations, while a score of 1 represents complete sharing of all *var* DBLα types

Observed number of shared DBLα types (PTS Score)	Colombia	Venezuela	Peru	French Guiana–Camopi	French Guiana– Trois Sauts
Colombia	112 (1.00)	—	—	—	—
Venezuela	36 (0.25)	176 (1.00)	—	—	—
Peru	32 (0.24)	64 (0.38)	157 (1.00)	—	—
French Guiana–Camopi	13 (0.08)	106 (0.52)	53 (0.28)	229 (1.00)	—
French Guiana–Trois Sauts	13 (0.08)	101 (0.52)	51 (0.28)	191 (0.87)	210 (1.00)

**Figure 5:**
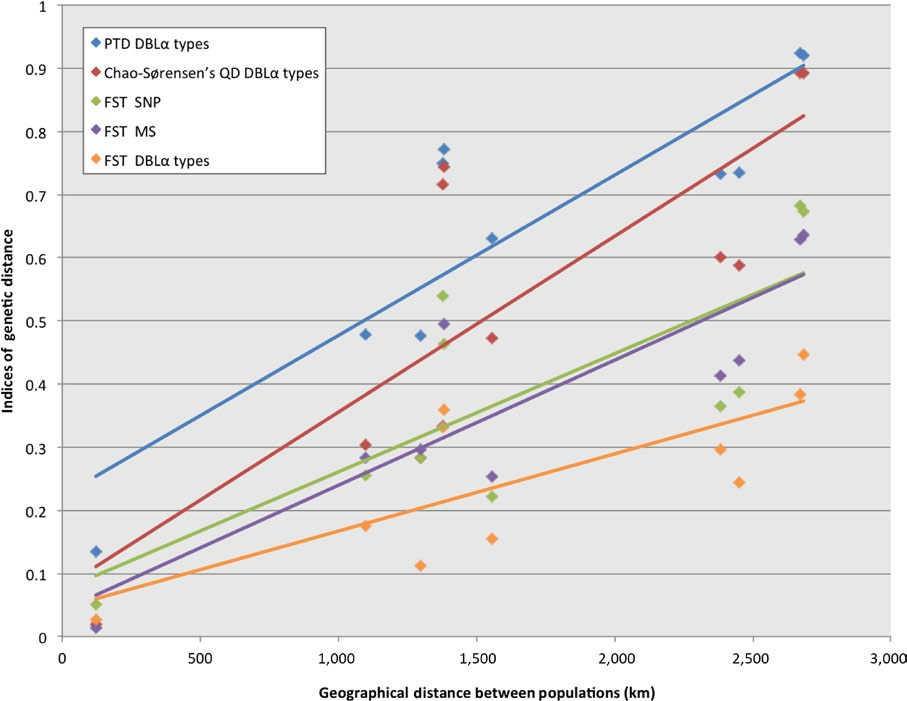
Isolation by distance between the South America populations. Pairwise geographic distances (km) between pairs of populations (indicated on the *x*‐axis) were plotted against the indices of genetic distance for each dataset: PTD *var* DBLα types, Chao–Sørensen's QD *var* DBLα types, *F*_ST_ SNP,*F*_ST_ MS, and *F*_ST_ DBLα types. The Pearson's correlation coefficients (*r*) were calculated between the various indices of genetic distance and were determined to be as follows: PTD *var* DBLα types (*r *= 0.88, blue), Chao–Sørensen's QD *var* DBLα types (*r *= 0.81, red), *F*_ST_ SNP (*r *= 0.77, green), *F*_ST_ MS (*r *= 0.88, purple), and *F*_ST_ *var* DBLα types (*r *= 0.76, orange)

To further understand this pattern of DBLα type sharing, comparisons were made to evaluate the effects of isolation by distance (IBD) between the South American populations. Figure 5 presents the pairwise geographic distances (in km, pairwise distance calculations available in Figure 1b) plotted against the various indices of genetic distance (*F*_ST_, PTD*, Chao–Sørensen's QD*) for SNPs, MS, and DBLα types. This analysis showed a positive correlation between geographic distance and the indices of genetic distance for the DBLα types (*r* values ≥ 0.76), and that these patterns were comparable to the other neutral markers (SNPs and MS) previously examined (Yalcindag et al., 2012). Increasing the geographic distance between the South American populations resulted in less genetic sharing being observed between isolates (Figure 5). Despite this trend, abundant DBLα types were conserved among all the South American populations, with 10 DBLα types (2.2%) being seen across all four populations surveyed, and 57 DBLα types (12.4%) being observed among three of the South American populations. When the indices of genetic distance (*F*_ST_) based on the SNPs and MS markers were compared to different indices of genetic distance (*F*_ST_, PTD*, Chao–Sørensen's QD*) for the DBLα types, positive correlations were observed (all *r* values were ≥0.92). Therefore, increasing either the genetic distance (*F*_ST_) for the SNPs or MS markers resulted in an increased genetic distance (*F*_ST_, PTD, *Chao–Sørensen's QD*) for the DBLα types (Fig. S5). This result indicates that both traditional neutral markers (SNPs and MS) and immune‐selected markers (DBLα types) can be used to examine genetic differentiation between populations in South America.

**Figure 6:**
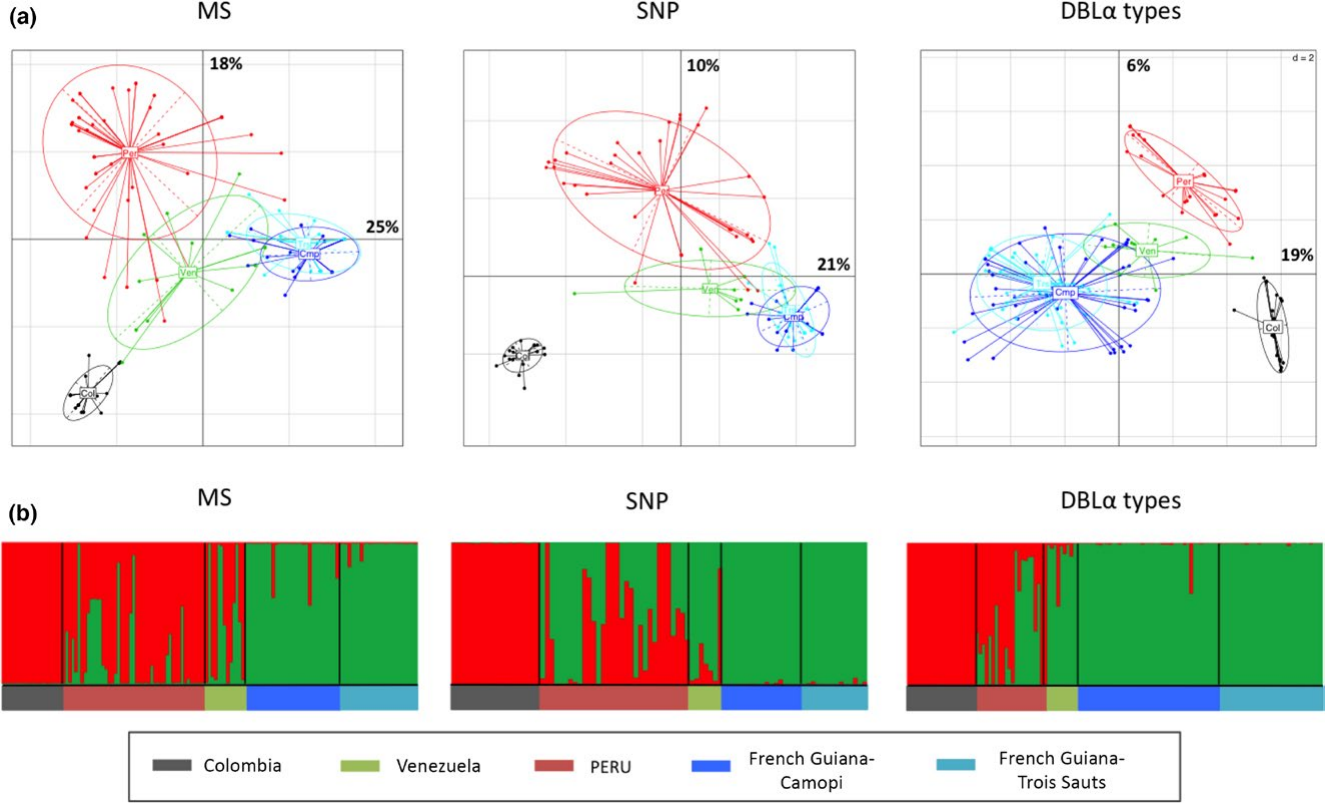
Genetic relationship between South American populations, based on MS, SNPs, and *var* DBLα types. (a) Principal component analysis, where the colored dots represent the population isolates, and the colored ellipses represent 95% of the genetic variation within each population. Percentages of inertia are displayed directly along the respective axes (first axis: horizontal; second axis: vertical). The code name of each population is at the centroid of the ellipse: Colombia (grey, Col), Venezuela (green, Ven), Peru (red, Per), French Guiana–Camopi (blue, Cmp), and French Guiana–Trois Sauts (turquoise, Trs). (b) South American population structure inferred by Bayesian clustering. Each isolate is a column and is partitioned into *K* colored components (*K* = 2). Boxes represent the assignment proportions to two clusters (*K* = 2), the optimal number of South American clusters inferred from the STRUCTURE simulations

Finally, the PCA based on the SNPs, MS, and the DBLα types (Figure 6a) clearly showed differentiation between isolates from Colombia, French Guiana (Camopi and Trois Sauts), Venezuela, and Peru. Moreover, these PCA results were consistent with the Bayesian clustering analysis (Figure 6b). Indeed, a clear separation was observed between the Colombian isolates and the other populations surveyed. By analyzing either the DBLα types, SNPs, or MS, the Bayesian clustering analyses suggested that the Peruvian parasite population, and to a lesser extent the Venezuelan population, were a mixture between the Colombian and French Guiana populations (Figure 6b). The admixed nature of the Peruvian and Venezuelan populations was further evident from the neighbor joining tree (Fig. S6).

## 4 DISCUSSION

Molecular surveillance of diverse pathogens like *P. falciparum* that are constantly evolving is critical to achieve control. In this context, detection of genetic variation in the genes encoding the major surface antigens is important for disease surveillance as immunity to such antigens drives the dynamics of the transmission system and allows for epidemics to be antigenically characterized. For example, the single copy hemagglutinin gene of the influenza virus is used to predict spatial and temporal patterns of disease (Munster et al., 2007; Nelson & Holmes, 2007; Plotkin, Dushoff, & Levin, 2002; Rambaut et al., 2008). In contrast, for *P. falciparum* monitoring antigenic diversity is very complex. Numerous polymorphic surface antigen encoding genes in different life cycle stages (e.g., *rif*,* stevor*,* msp1*,* msp2*) have been utilized as diagnostic markers of diversity (Baruch et al., 1995; Bull et al., 2005; Cheng et al., 1998; Florens et al., 2002; Gardner et al., 2002; Sam‐Yellowe et al., 2004; Scherf et al., 2008; Smith et al., 1995; Su et al., 1995; Woehlbier et al., 2010). Specifically, the major *P. falciparum* variant surface antigen of the blood stages, *Pf*EMP1, is a key marker as it is a virulence factor and immunity to this antigen determines the dynamics of infection within and between hosts (e.g., (Artzy‐Randrup et al., 2012)). To date few malaria surveillance studies have used *var* genes encoding *Pf*EMP1 due to the extreme diversity and the complexity of undertaking population genetics with this multigene family (Albrecht et al., 2010; Artzy‐Randrup et al., 2012; Chen et al., 2011; Day et al., 2017; Tessema et al., 2015). Here, using the 454 high throughput sequencing approach to obtain well‐sampled populations, we show that the conserved DBLα domain of *var* genes constitutes a promising biomarker to infer population structure, and more generally for epidemiological disease surveillance. Indeed, we demonstrate that DBLα types were spatially variable and geographically structured in South America.

In contrast to *P. falciparum* in Africa, we observed “limited” *var* DBLα type diversity within the local South American populations, which was consistent with previous surveys (Albrecht et al., 2010; Chen et al., 2011). One hypothesis to explain this “limited” *var* DBLα type diversity could be linked to relatively lower transmission and hence fewer co‐infections/superinfections (i.e., multiple genotypes within an isolate) existing in the South American human population. This would lead to less frequent outcrossing (mating between two genetically distinct parasites) during meiotic recombination in the mosquito phase of the parasite life cycle. The observed limited local diversity of DBLα types in South America raises the possibility that the highly immunogenic *Pf*EMP1 could be a vaccine target in this location.

The South American east/west geographic differentiation in *var* DBLα types mirrors the population structure reported previously using SNPs and MS markers in the same populations (Yalcindag et al., 2012). Like the neutral/non‐selected markers, *var* DBLα type structuring was also consistent with an isolation‐by‐distance model. The significant genetic differentiation obtained could be explained by the “limited” genetic diversity observed in the populations under study (Hedrick, 2005), the epidemic characteristic and small effective size of South American *P. falciparum* populations (Anderson et al., 2000) as well as multiple independent introductions of *P. falciparum* into South America (Yalcindag et al., 2012).

Whether the underlying driver of geospatial structuring of the *var* loci is due to regional adaptation of *P. falciparum* genomes to unique mosquito vectors and/or to human demography/population history remains to be further examined. If the latter, then we suggest that epidemic transmission of *P. falciparum* could occur across South America through the importation of novel variants not previously seen in the region. Indeed, the low shared diversity (few *var* DBLα types) and/or mixing among the South American countries suggests the necessity to develop local strategies for vaccination *vs*. a pan‐continental approach. To conclude, the use of *var* population genomics in South America allowed for the description of the genetic complexity in the reservoir of infection as well as a better understanding of *P. falciparum* epidemiology in relation to differences in parasite antigenic variation.

## CONFLICT OF INTEREST

None declared.

## DATA ACCESSIBILITY

All DBLα sequences analyzed in this study are publicly available for download in GenBank: KX845707–KX851405.
