# Supplementary material for: Evolutionary structure of Plasmodium falciparum major variant surface antigen genes in South America: Implications for epidemic transmission and surveillance
Source: Ecol Evol. 2017 Oct 8;7(22):9376–90. doi: 10.1002/ece3.3425 (PMC5696401; doi:10.1002/ece3.3425)
Supplement: Supplementary file 1 [file ECE3-7-9376-s001.pdf]

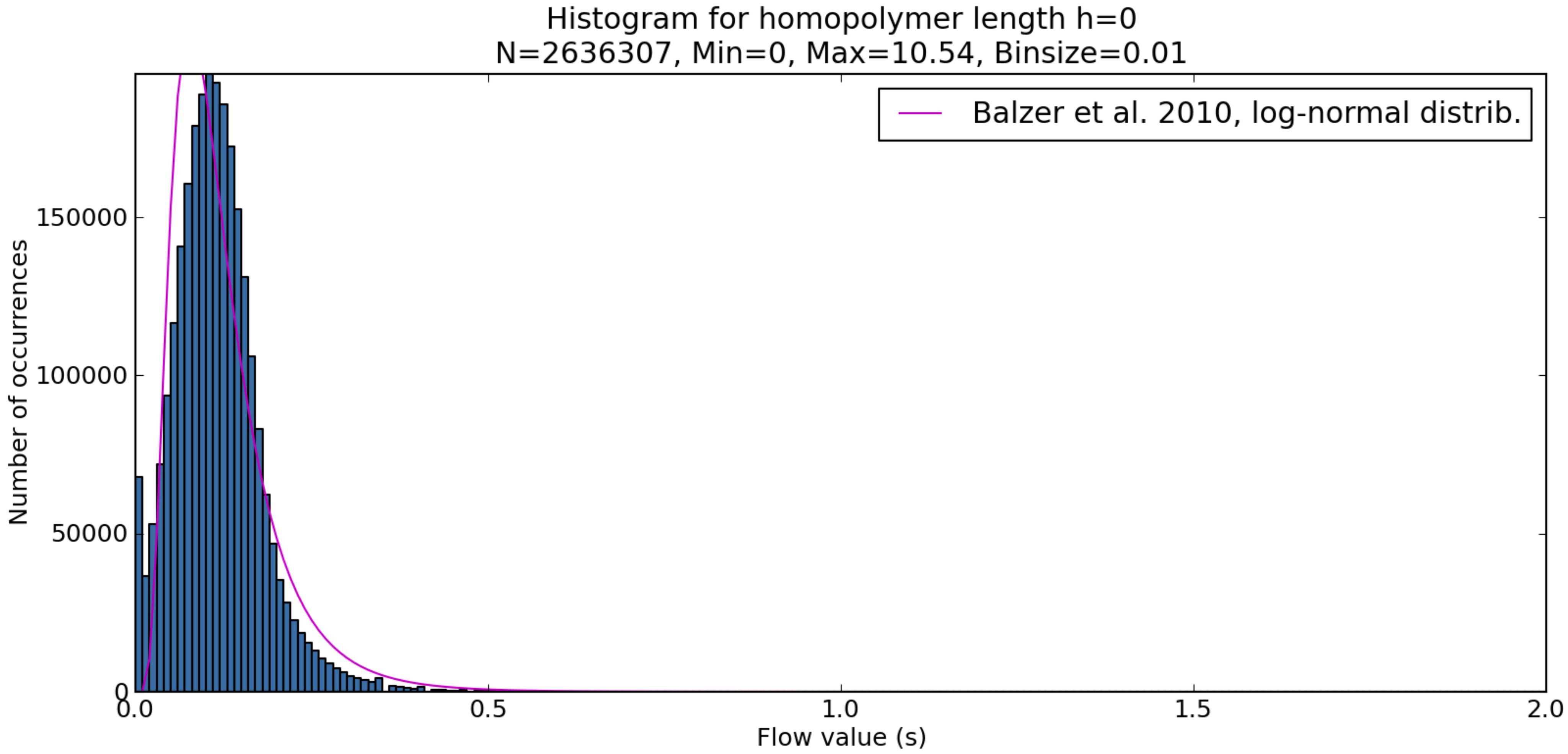

Histogram for homopolymer length h=0
N=2636307, Min=0, Max=10.54, Binsize=0.01
Balzer et al. 2010, log-normal distrib.
Number of occurrences
0
50000
100000
150000
0.0
0.5
1.0
1.5
2.0
Flow value (s)

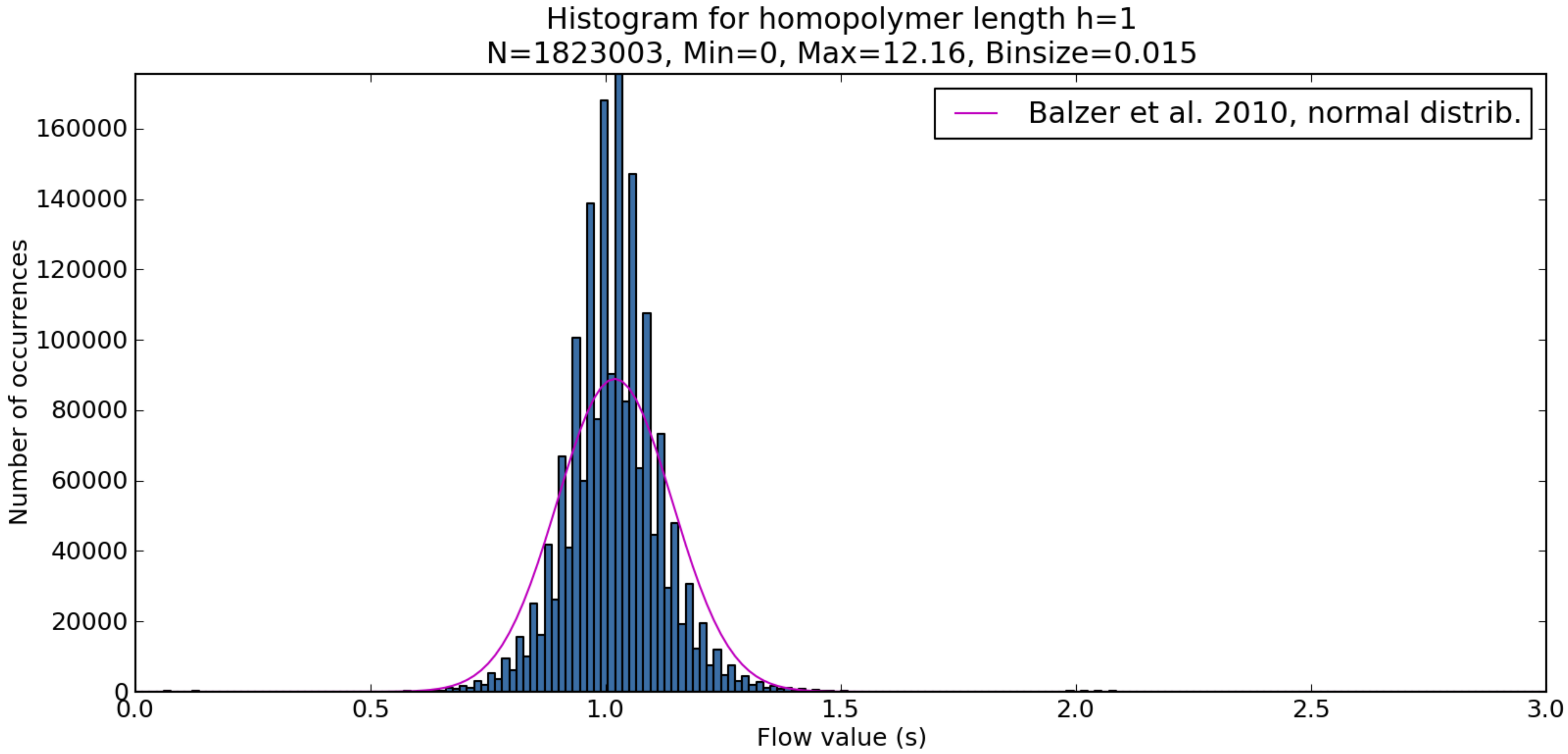
Histogram for homopolymer length h=1
N=1823003, Min=0, Max=12.16, Binsize=0.015
Balzer et al. 2010, normal distrib.
Number of occurrences
Flow value (s)
0
20000
40000
60000
80000
100000
120000
140000
160000
0.0
0.5
1.0
1.5
2.0
2.5
3.0

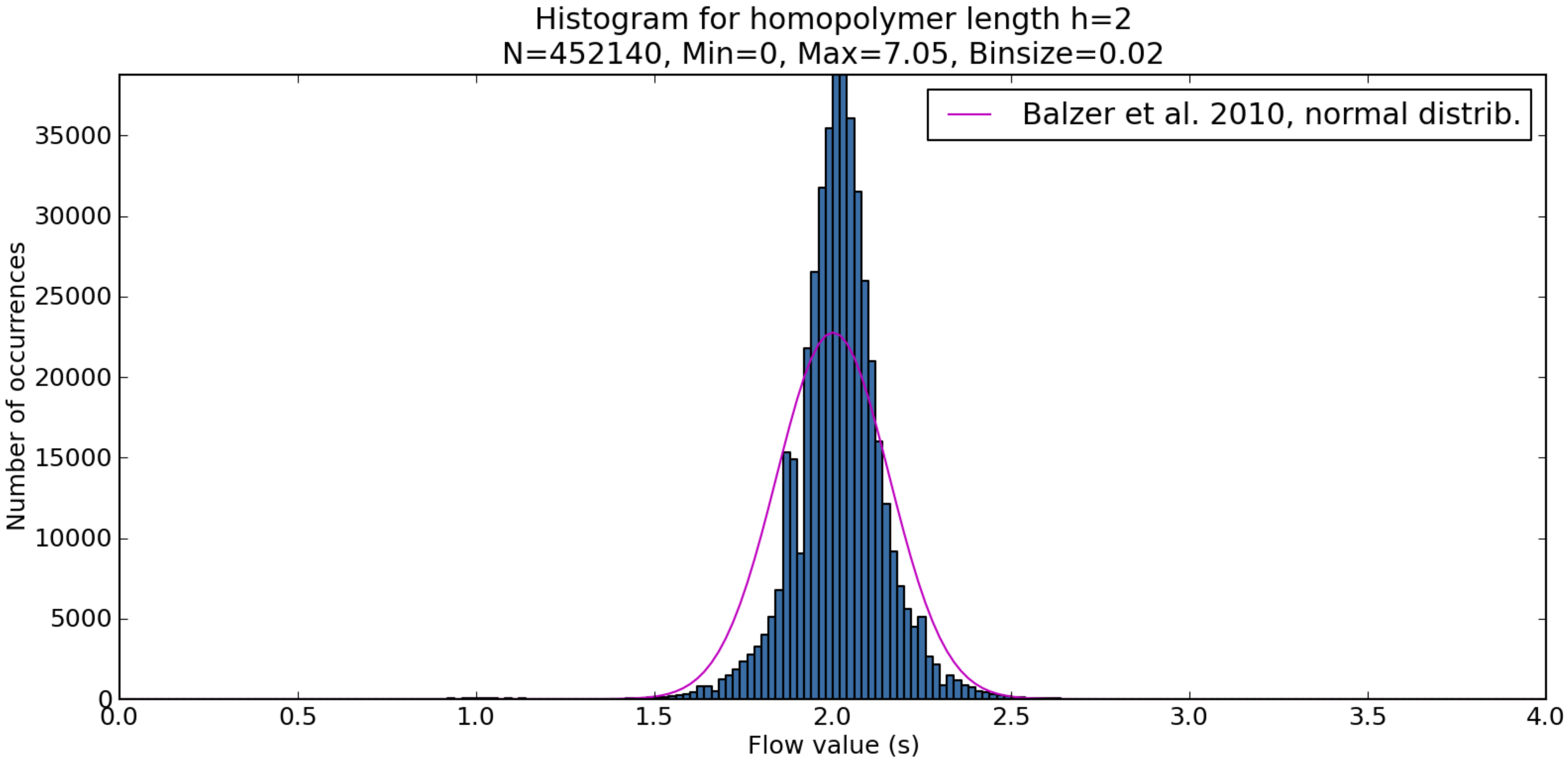

Histogram for homopolymer length h=2
N=452140, Min=0, Max=7.05, Binsize=0.02
Balzer et al. 2010, normal distrib.
Number of occurrences
Flow value (s)
0
5000
10000
15000
20000
25000
30000
35000
0.0
0.5
1.0
1.5
2.0
2.5
3.0
3.5
4.0

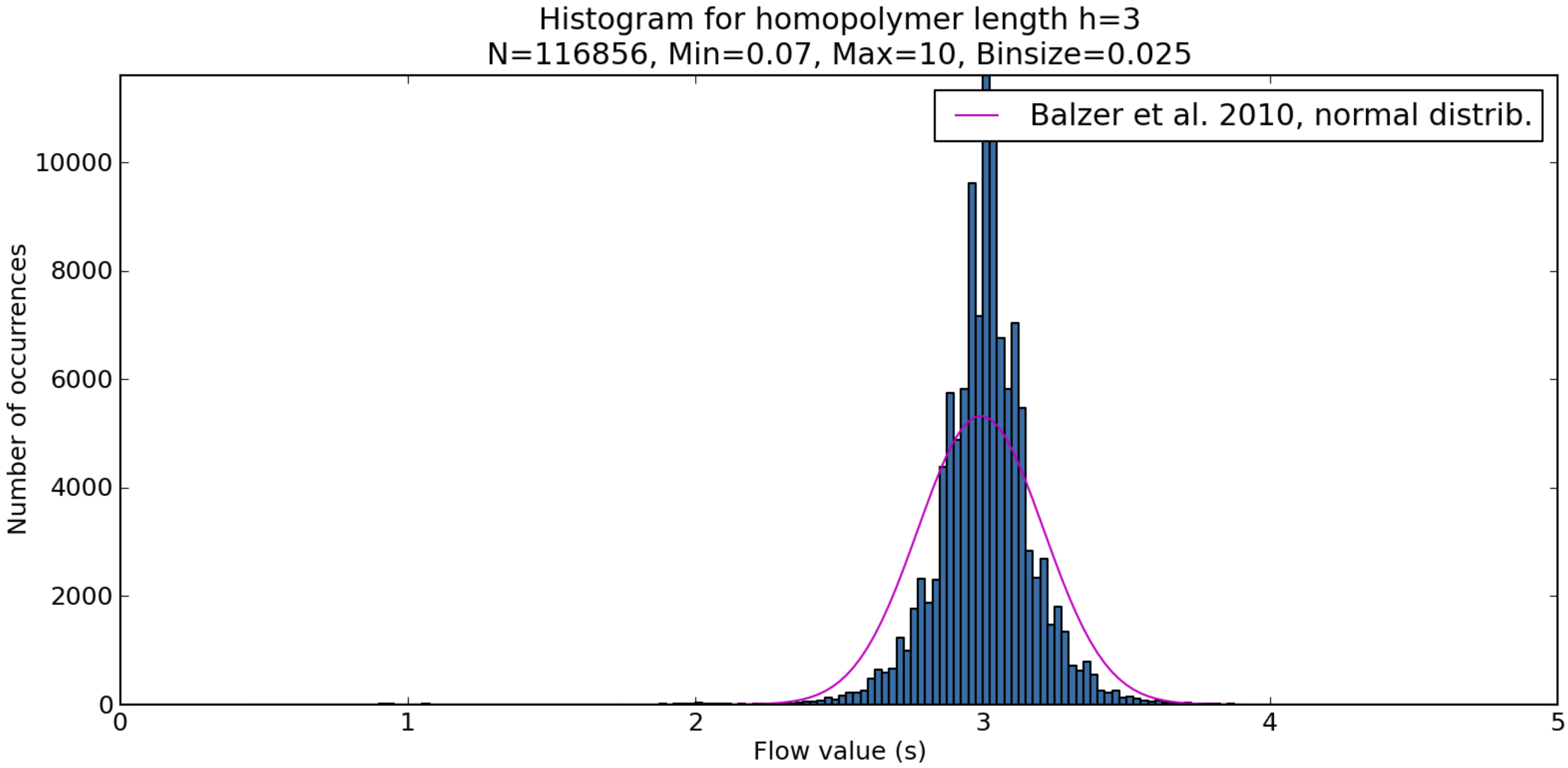
Histogram for homopolymer length h=3
N=116856, Min=0.07, Max=10, Binsize=0.025
Balzer et al. 2010, normal distrib.
Number of occurrences
10000
8000
6000
4000
2000
0
0
1
2
3
4
5
Flow value (s)

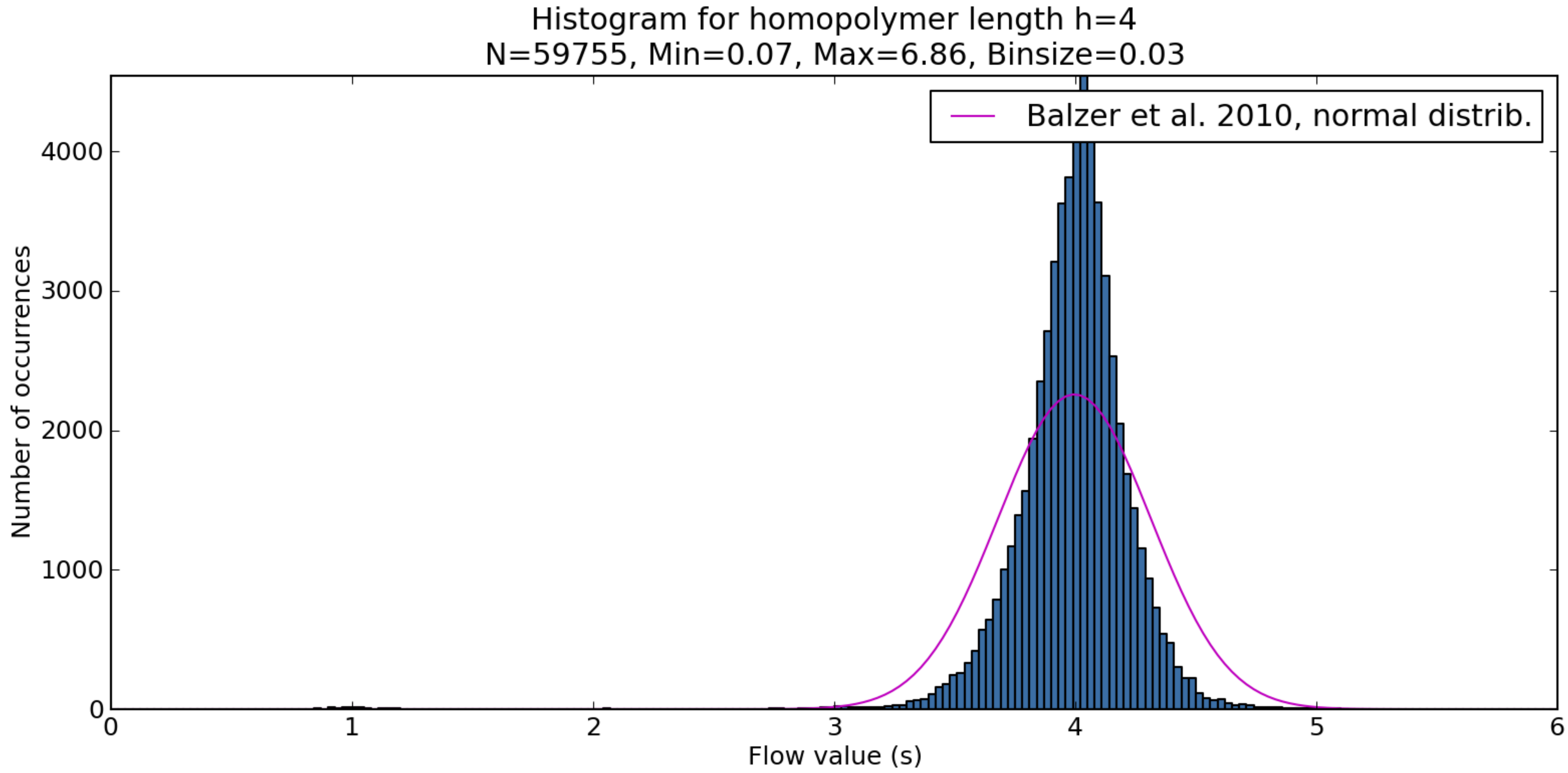
Histogram for homopolymer length h=4
N=59755, Min=0.07, Max=6.86, Binsize=0.03
Balzer et al. 2010, normal distrib.
Number of occurrences
Flow value (s)
0
1000
2000
3000
4000
0
1
2
3
4
5
6

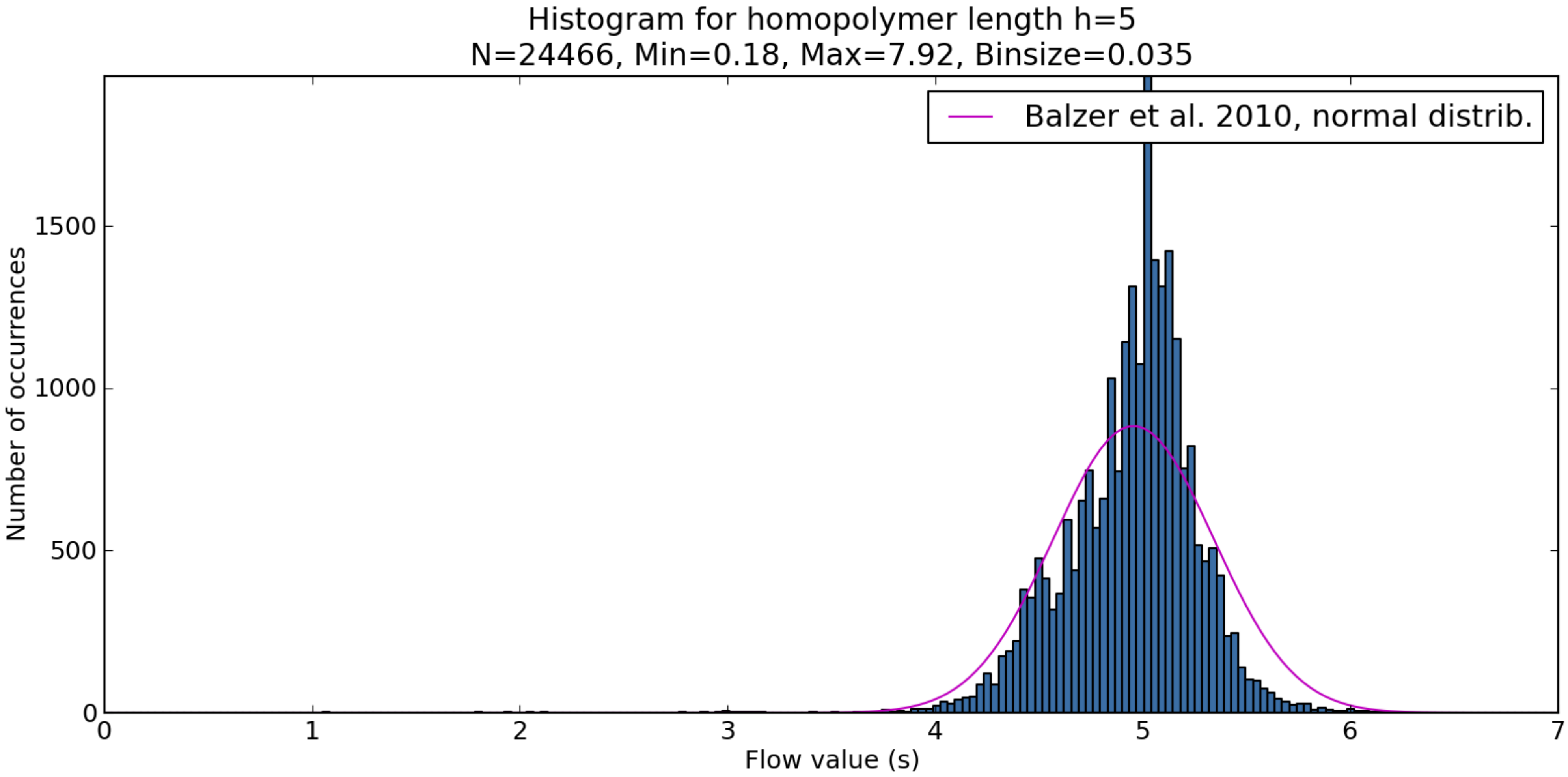
Histogram for homopolymer length h=5
N=24466, Min=0.18, Max=7.92, Binsize=0.035
Balzer et al. 2010, normal distrib.
Number of occurrences
Flow value (s)
0
500
1000
1500
0
1
2
3
4
5
6
7

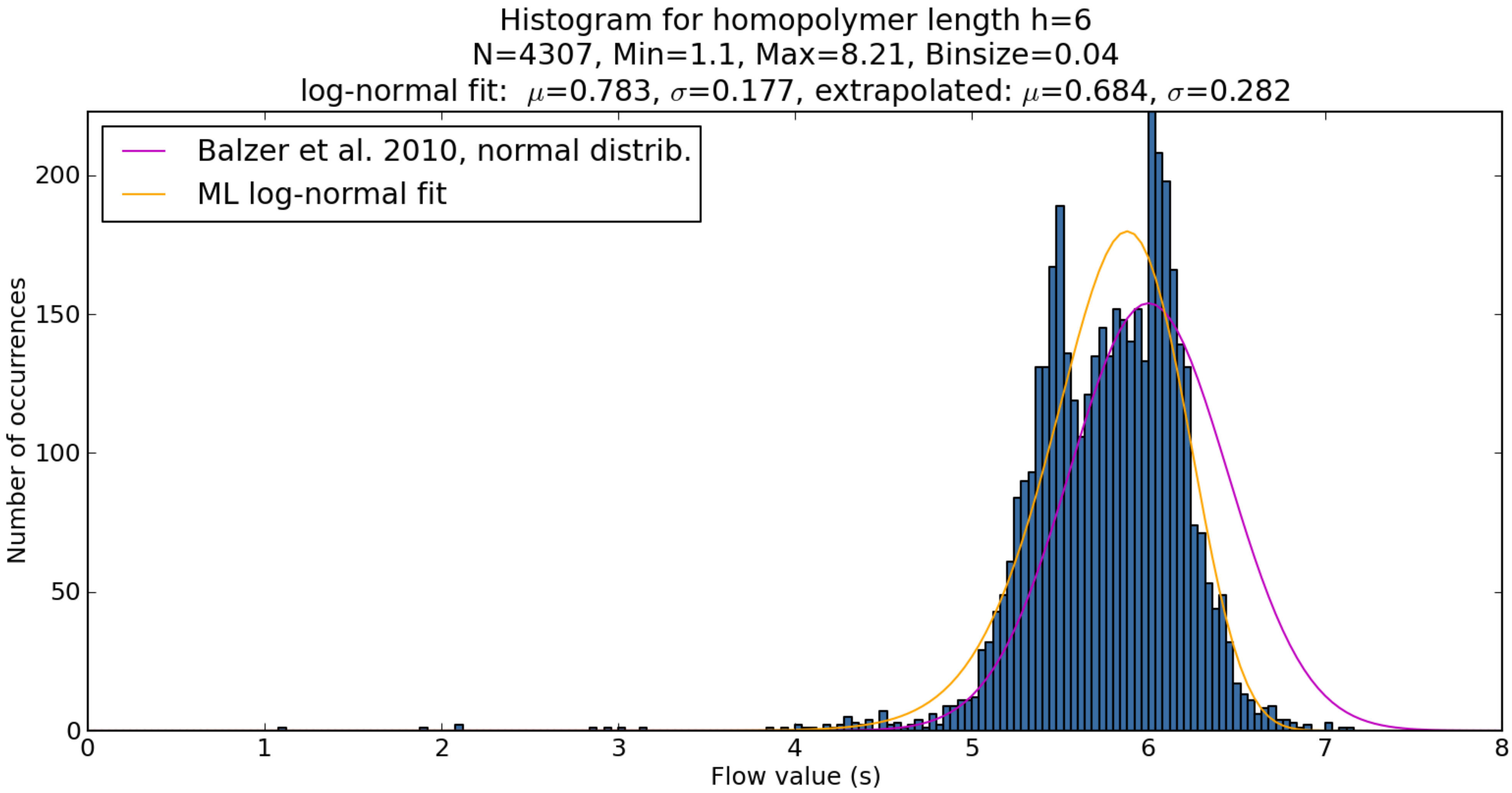

Histogram for homopolymer length h=6
N=4307, Min=1.1, Max=8.21, Binsize=0.04
log-normal fit: μ=0.783, σ=0.177, extrapolated: μ=0.684, σ=0.282
Balzer et al. 2010, normal distrib.
ML log-normal fit
Number of occurrences
Flow value (s)
0
50
100
150
200
0
1
2
3
4
5
6
7
8

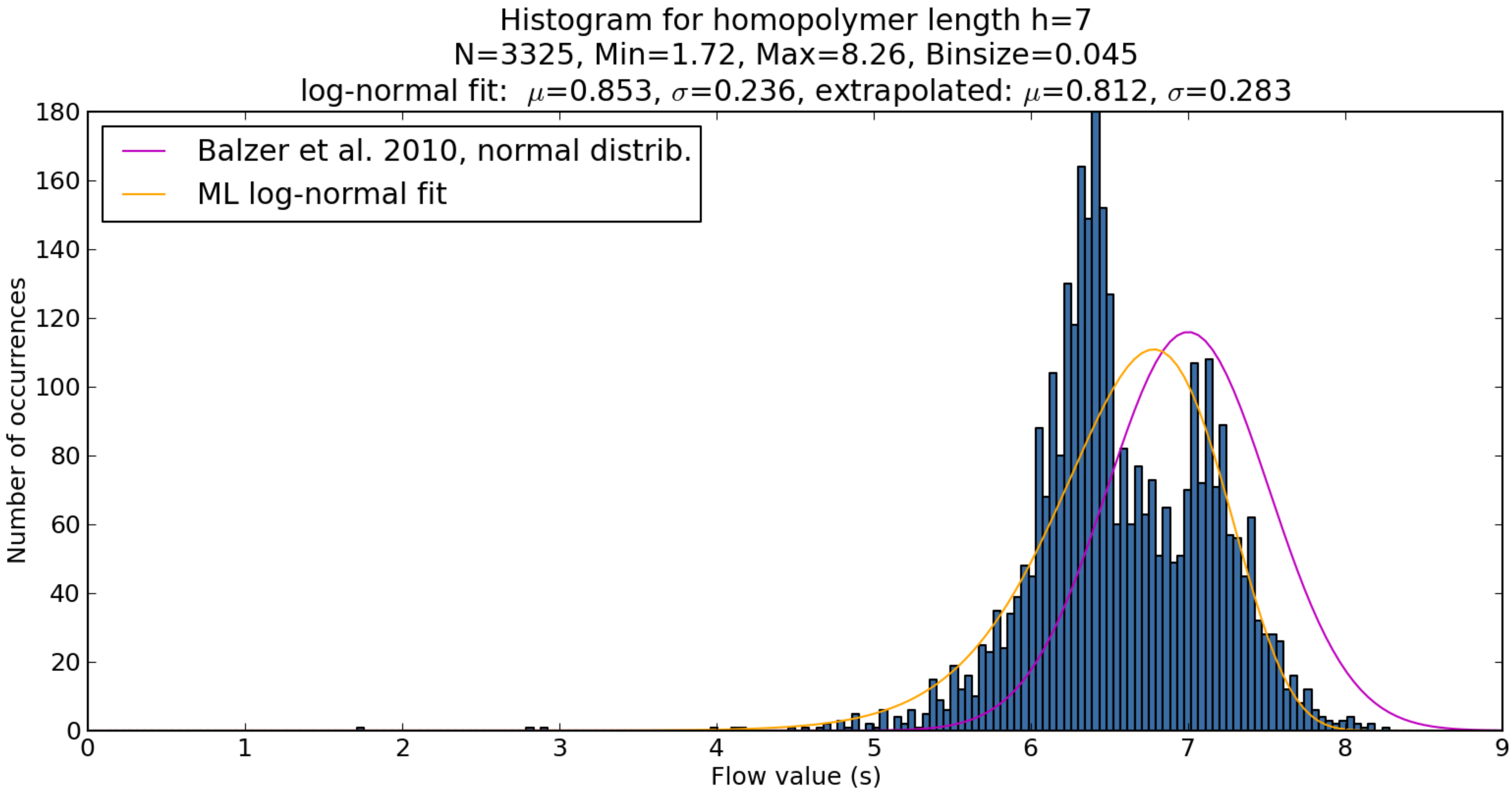
Histogram for homopolymer length h=7
N=3325, Min=1.72, Max=8.26, Binsize=0.045
log-normal fit: μ=0.853, σ=0.236, extrapolated: μ=0.812, σ=0.283
Balzer et al. 2010, normal distrib.
ML log-normal fit
Number of occurrences
Flow value (s)
0
20
40
60
80
100
120
140
160
180
1
2
3
4
5
6
7
8
9

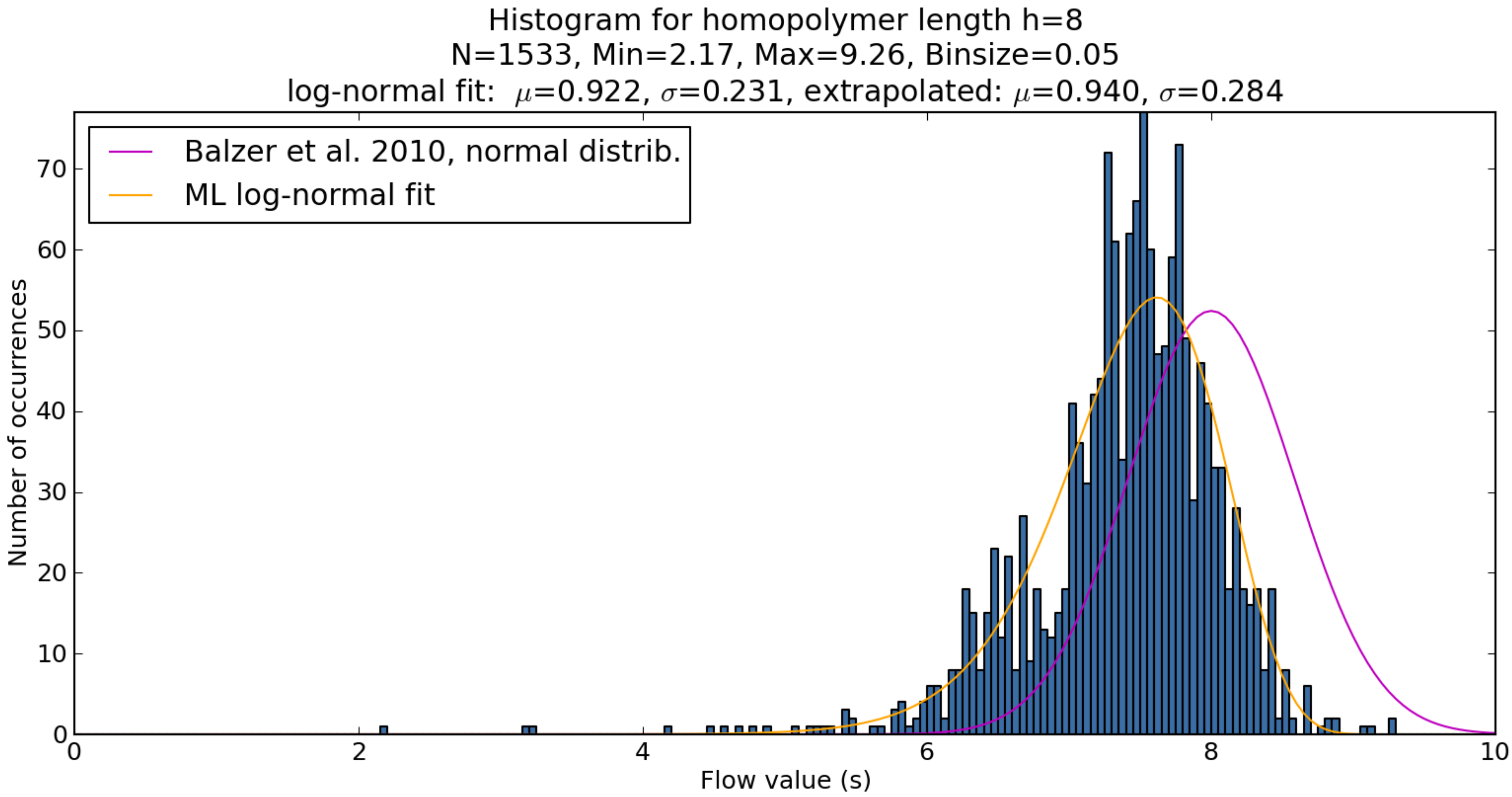
Histogram for homopolymer length h=8
N=1533, Min=2.17, Max=9.26, Binsize=0.05
log-normal fit: μ=0.922, σ=0.231, extrapolated: μ=0.940, σ=0.284
Balzer et al. 2010, normal distrib.
ML log-normal fit
Number of occurrences
Flow value (s)
0
10
20
30
40
50
60
70
0
2
4
6
8
10

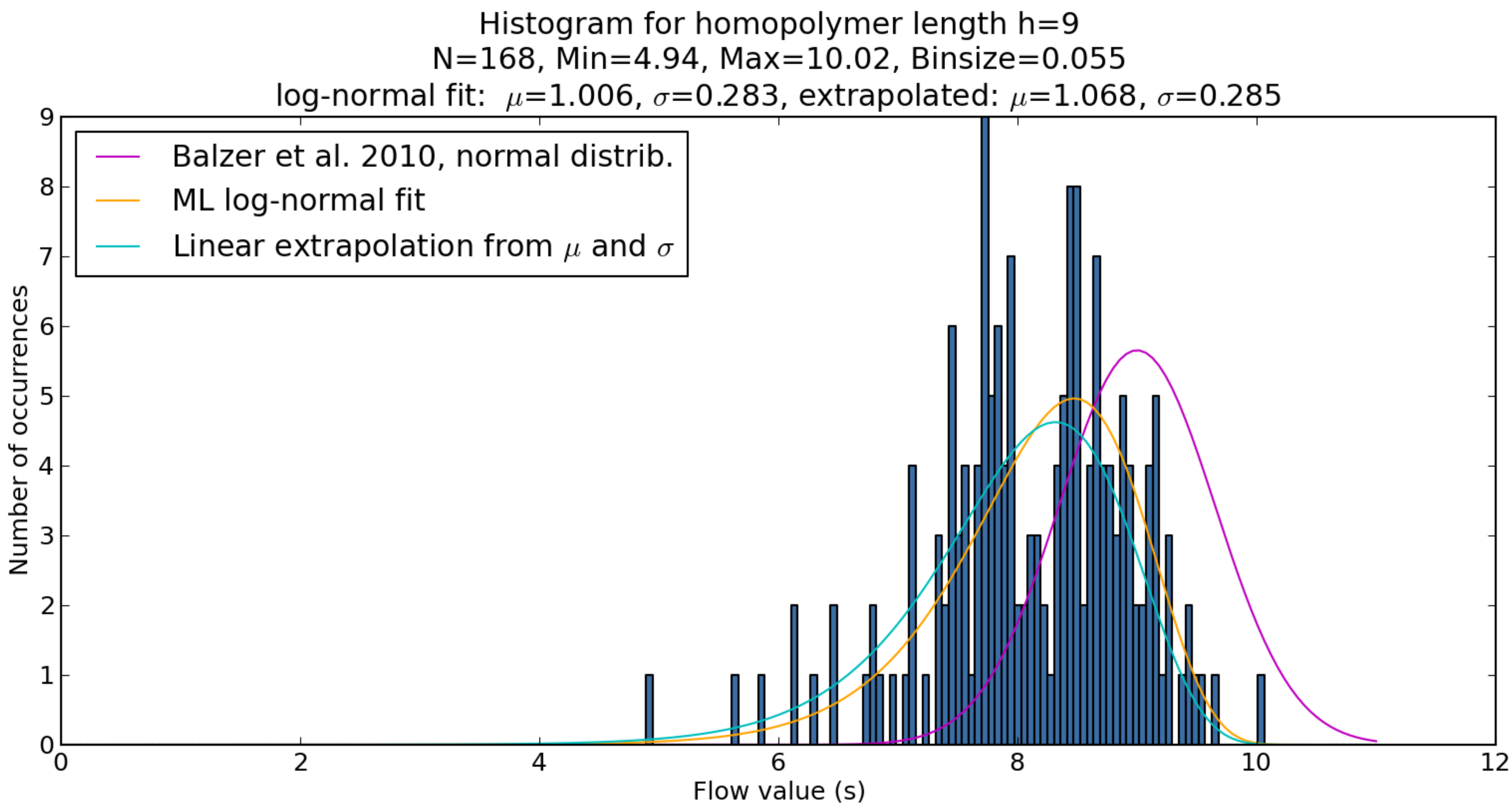
Histogram for homopolymer length h=9
N=168, Min=4.94, Max=10.02, Binsize=0.055
log-normal fit: μ=1.006, σ=0.283, extrapolated: μ=1.068, σ=0.285
Balzer et al. 2010, normal distrib.
ML log-normal fit
Linear extrapolation from μ and σ
Number of occurrences
Flow value (s)
0
1
2
3
4
5
6
7
8
9
0
2
4
6
8
10
12

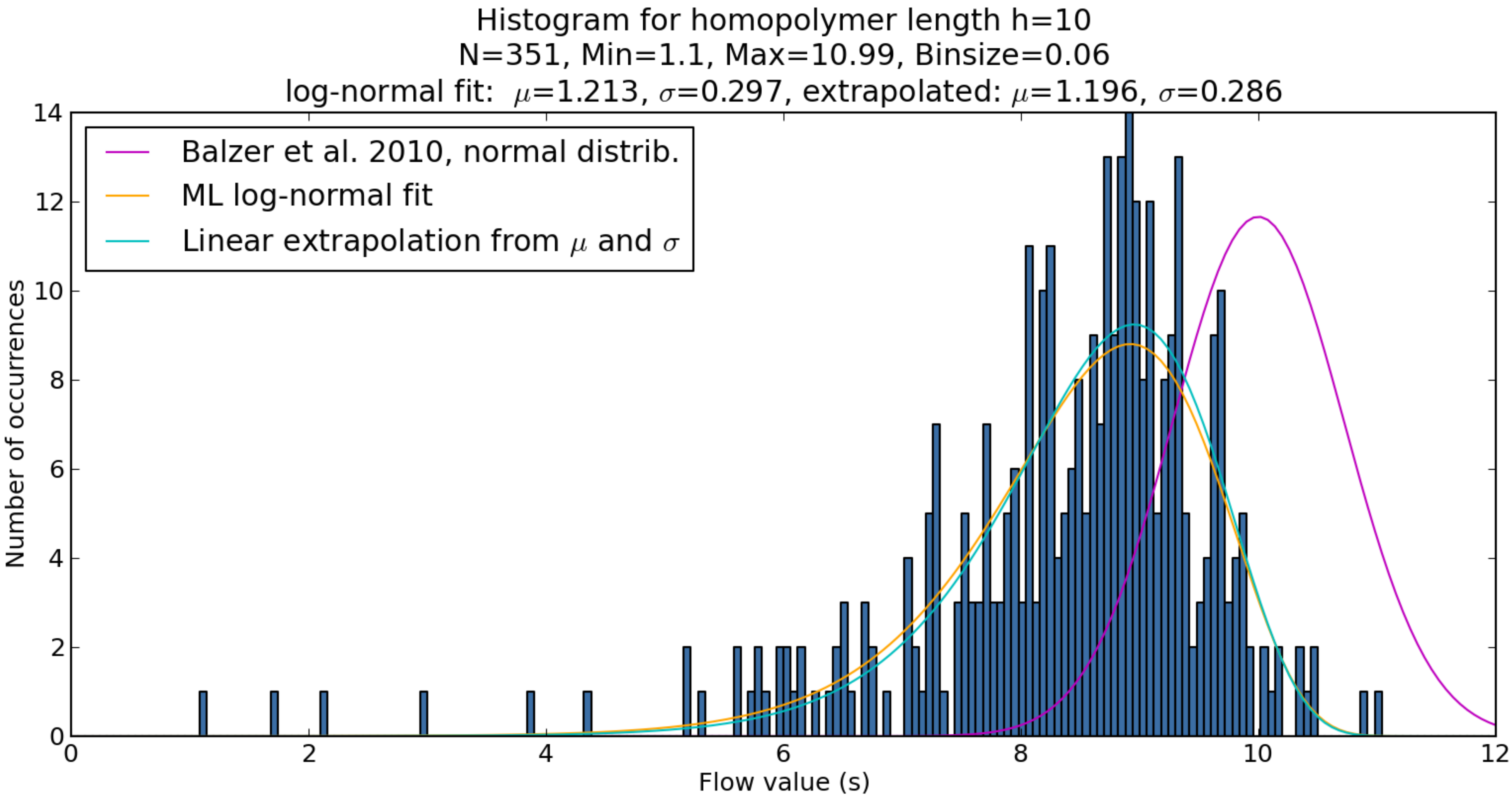
Histogram for homopolymer length h=10
N=351, Min=1.1, Max=10.99, Binsize=0.06
log-normal fit: μ=1.213, σ=0.297, extrapolated: μ=1.196, σ=0.286
Balzer et al. 2010, normal distrib.
ML log-normal fit
Linear extrapolation from μ and σ
Number of occurrences
Flow value (s)
0
2
4
6
8
10
12
14

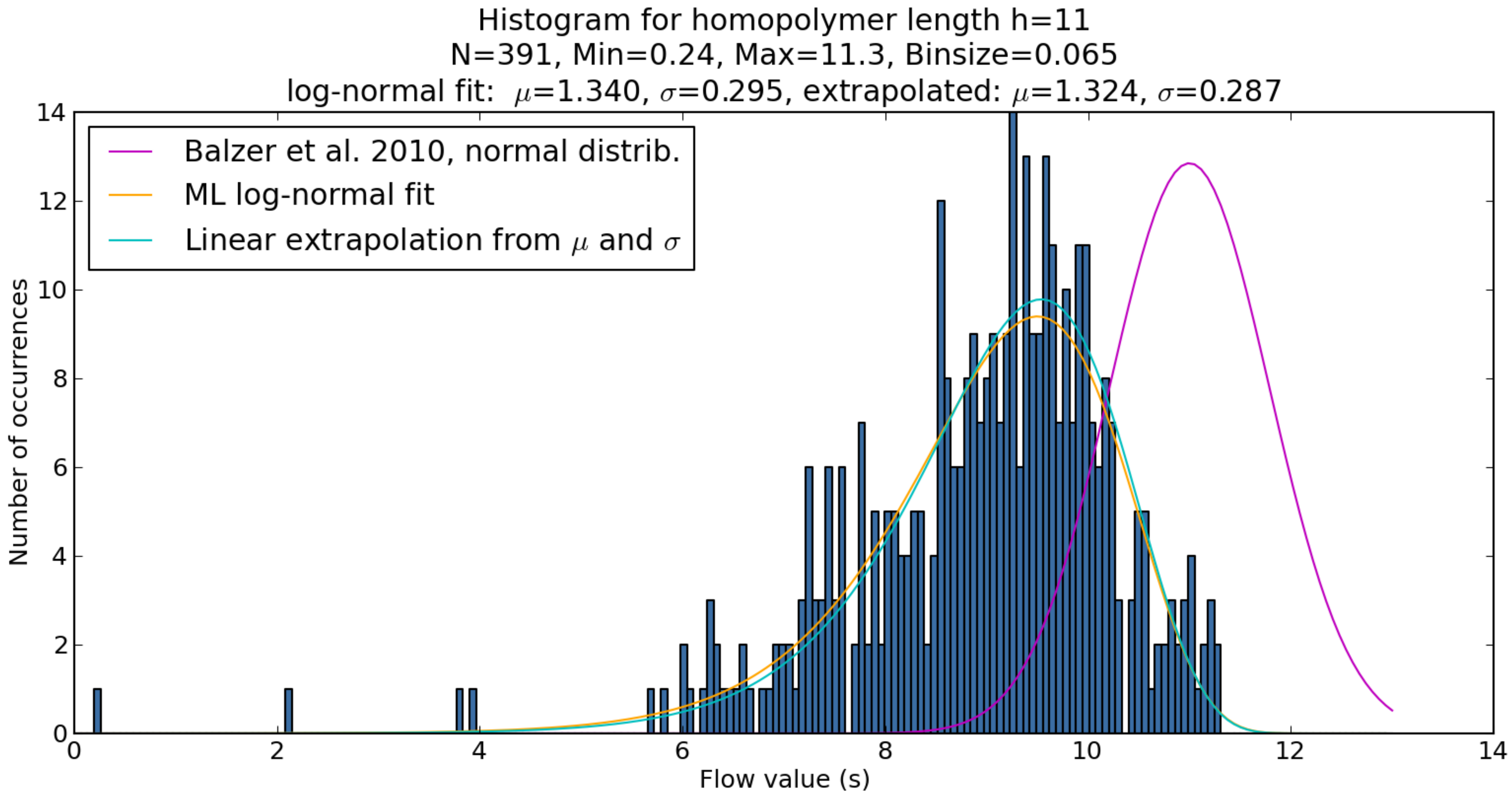

Histogram for homopolymer length h=11
N=391, Min=0.24, Max=11.3, Binsize=0.065
log-normal fit: μ=1.340, σ=0.295, extrapolated: μ=1.324, σ=0.287
Balzer et al. 2010, normal distrib.
ML log-normal fit
Linear extrapolation from μ and σ
Number of occurrences
Flow value (s)
0
2
4
6
8
10
12
14

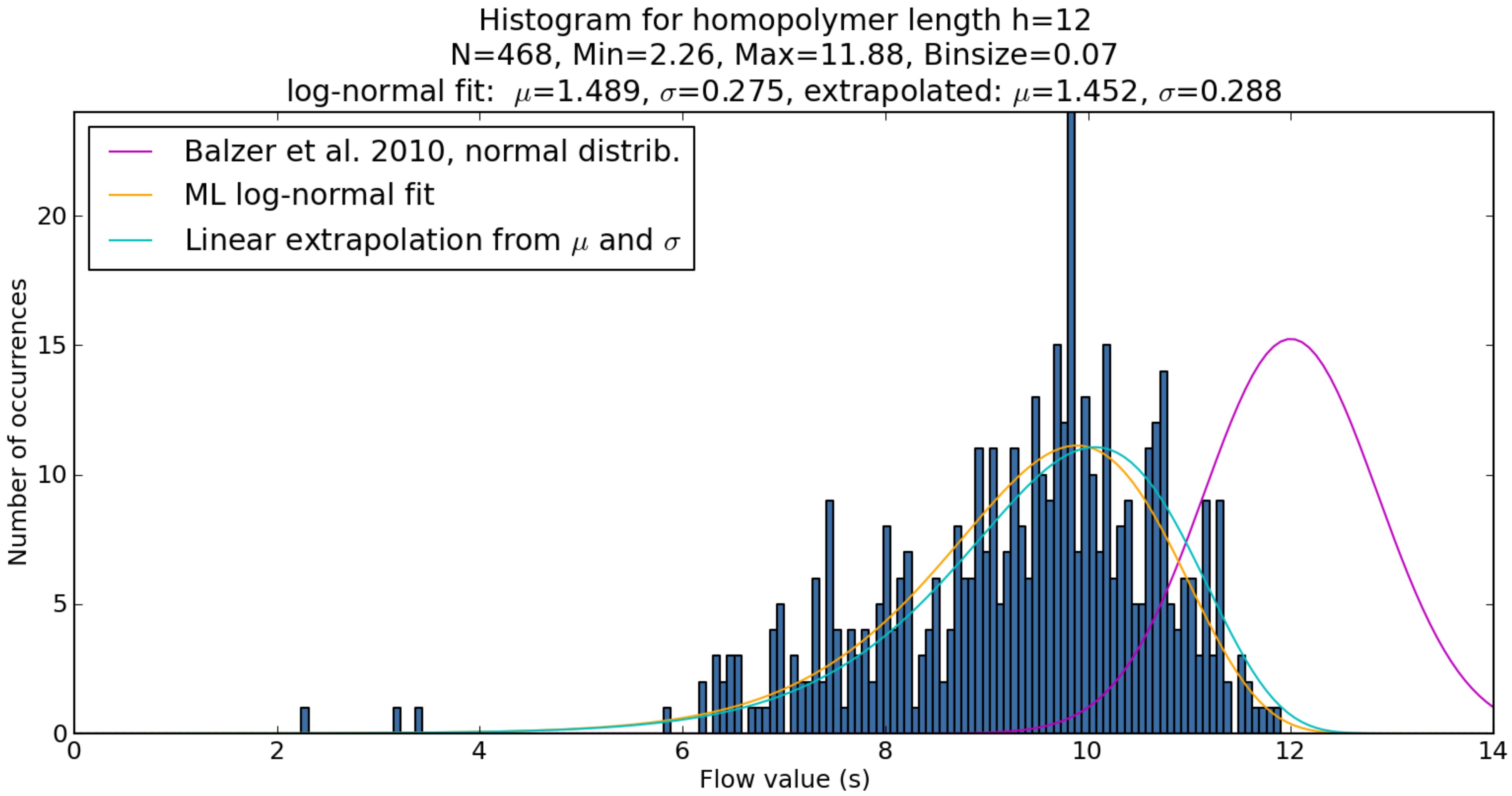
Histogram for homopolymer length h=12
N=468, Min=2.26, Max=11.88, Binsize=0.07
log-normal fit: μ=1.489, σ=0.275, extrapolated: μ=1.452, σ=0.288
Balzer et al. 2010, normal distrib.
ML log-normal fit
Linear extrapolation from μ and σ
Number of occurrences
Flow value (s)
0
5
10
15
20
0
2
4
6
8
10
12
14

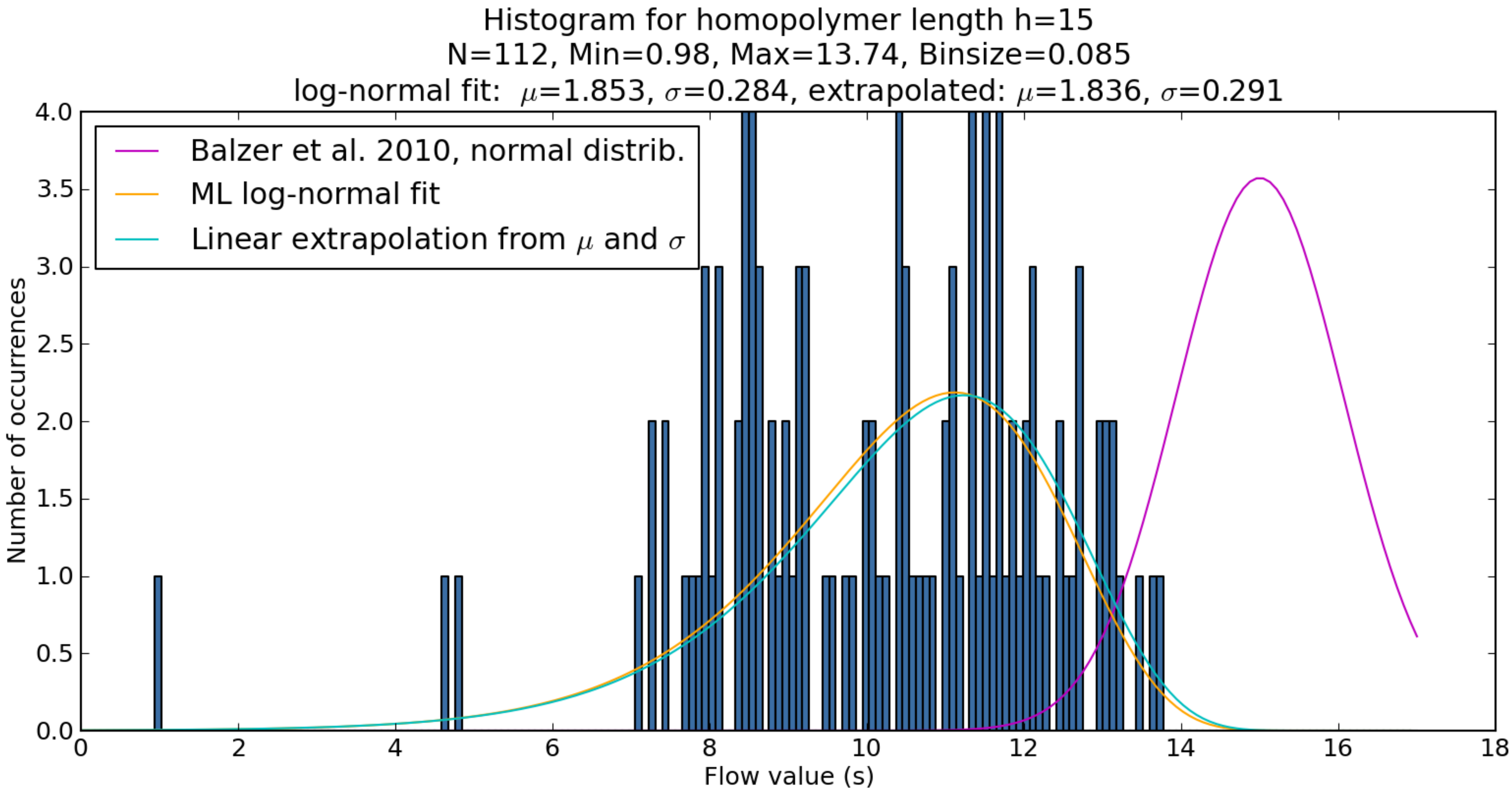
Histogram for homopolymer length h=15
N=112, Min=0.98, Max=13.74, Binsize=0.085
log-normal fit: μ=1.853, σ=0.284, extrapolated: μ=1.836, σ=0.291
Balzer et al. 2010, normal distrib.
ML log-normal fit
Linear extrapolation from μ and σ
Number of occurrences
Flow value (s)
0.0
0.5
1.0
1.5
2.0
2.5
3.0
3.5
4.0
0
2
4
6
8
10
12
14
16
18

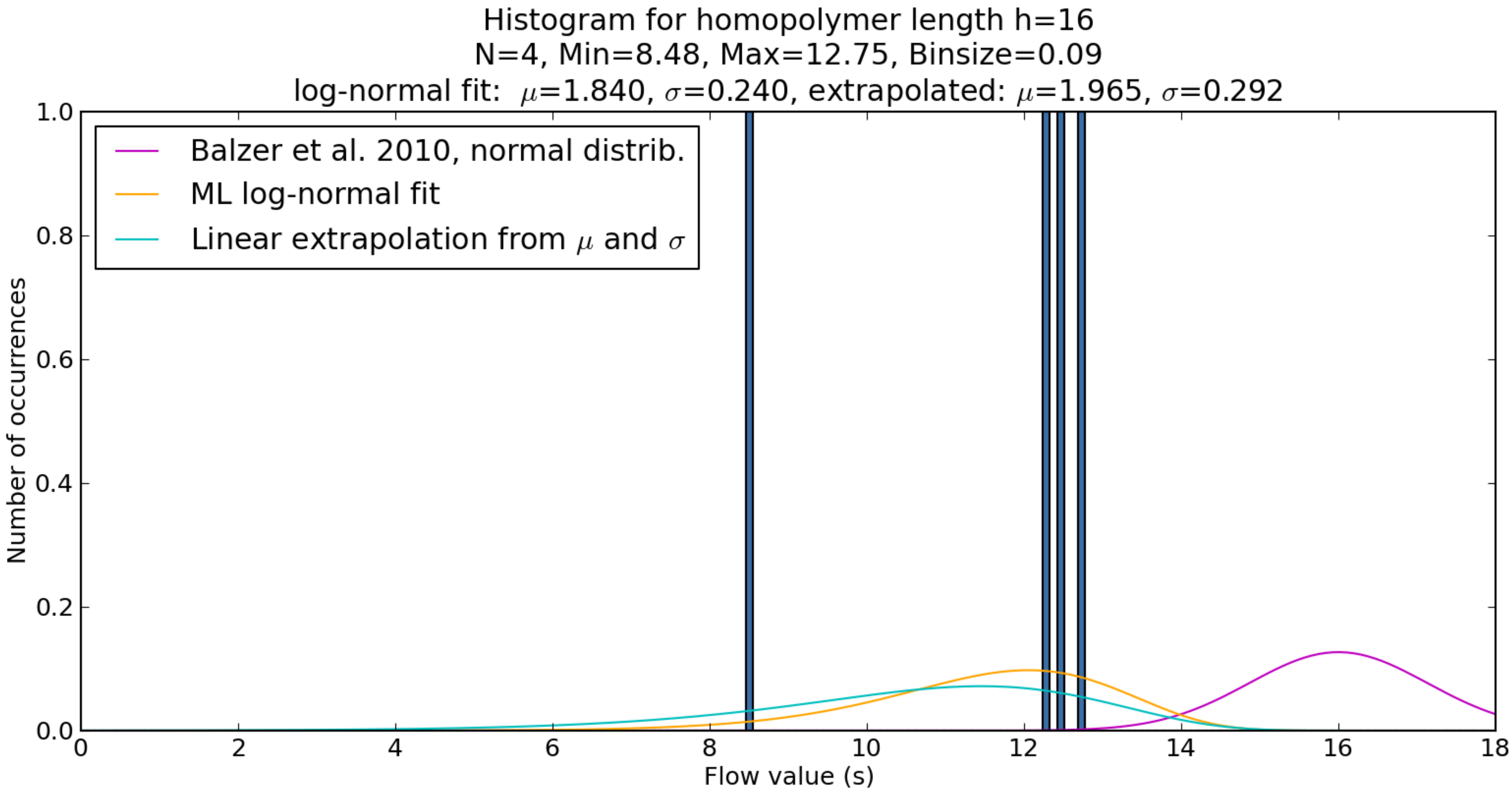

Histogram for homopolymer length h=16
N=4, Min=8.48, Max=12.75, Binsize=0.09
log-normal fit: μ=1.840, σ=0.240, extrapolated: μ=1.965, σ=0.292
Balzer et al. 2010, normal distrib.
ML log-normal fit
Linear extrapolation from μ and σ
Number of occurrences
Flow value (s)
1.0
0.8
0.6
0.4
0.2
0.0
0
2
4
6
8
10
12
14
16
18

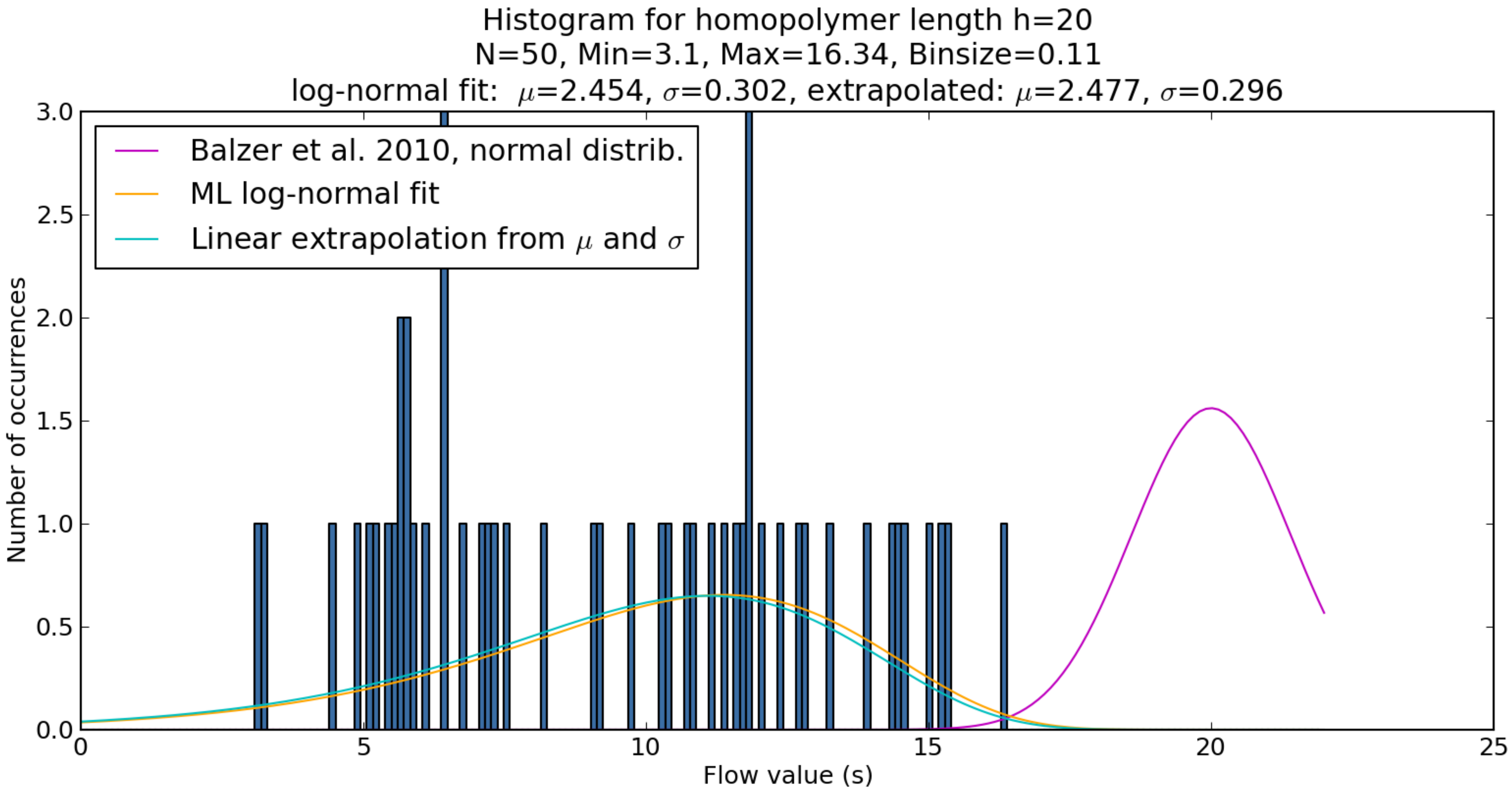

Histogram for homopolymer length h=20
N=50, Min=3.1, Max=16.34, Binsize=0.11
log-normal fit: μ=2.454, σ=0.302, extrapolated: μ=2.477, σ=0.296
Balzer et al. 2010, normal distrib.
ML log-normal fit
Linear extrapolation from μ and σ
Number of occurrences
Flow value (s)
0.0
0.5
1.0
1.5
2.0
2.5
3.0
0
5
10
15
20
25

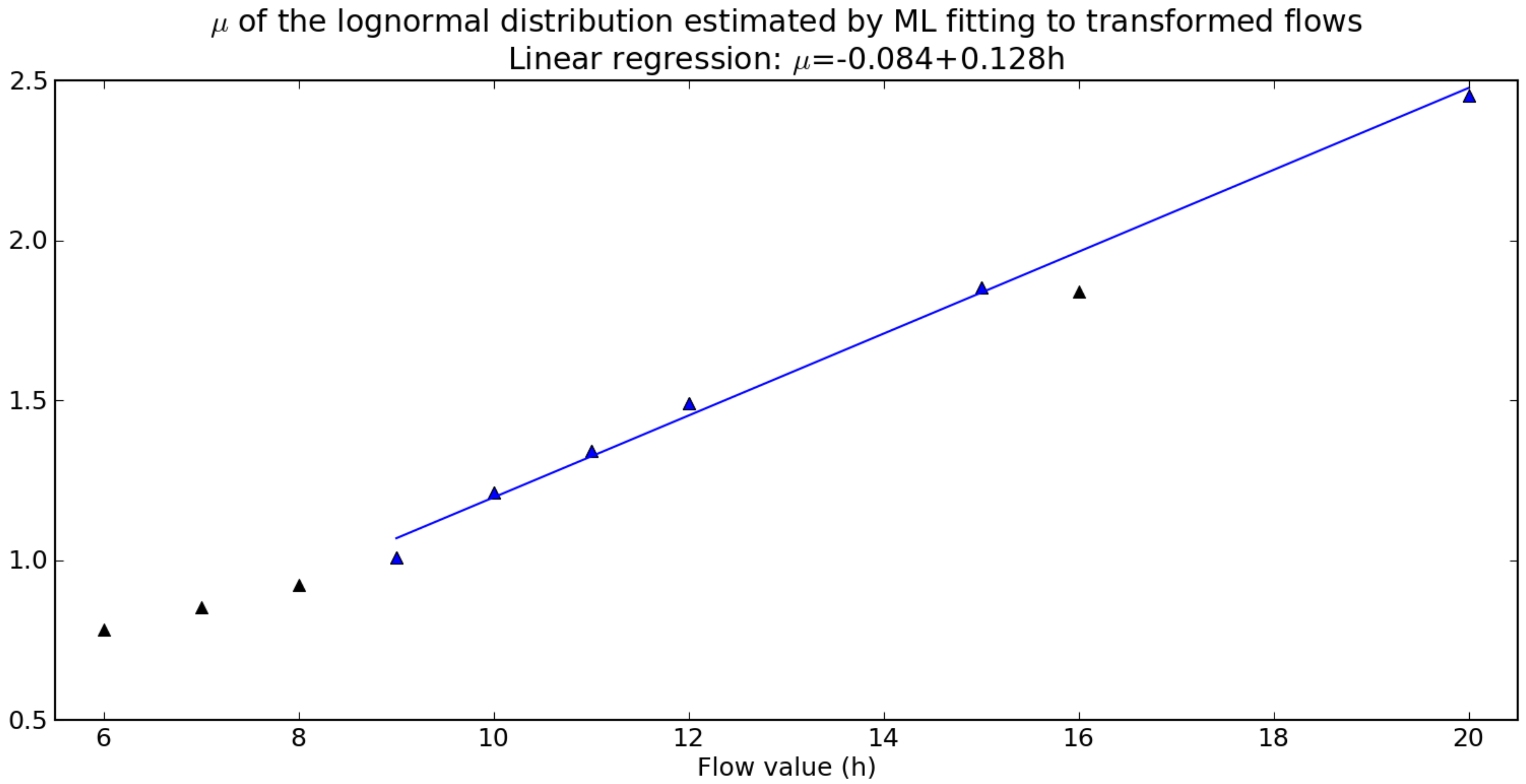
μ of the lognormal distribution estimated by ML fitting to transformed flows
Linear regression: μ=-0.084+0.128h
2.5
2.0
1.5
1.0
0.5
6
8
10
12
14
16
18
20
Flow value (h)

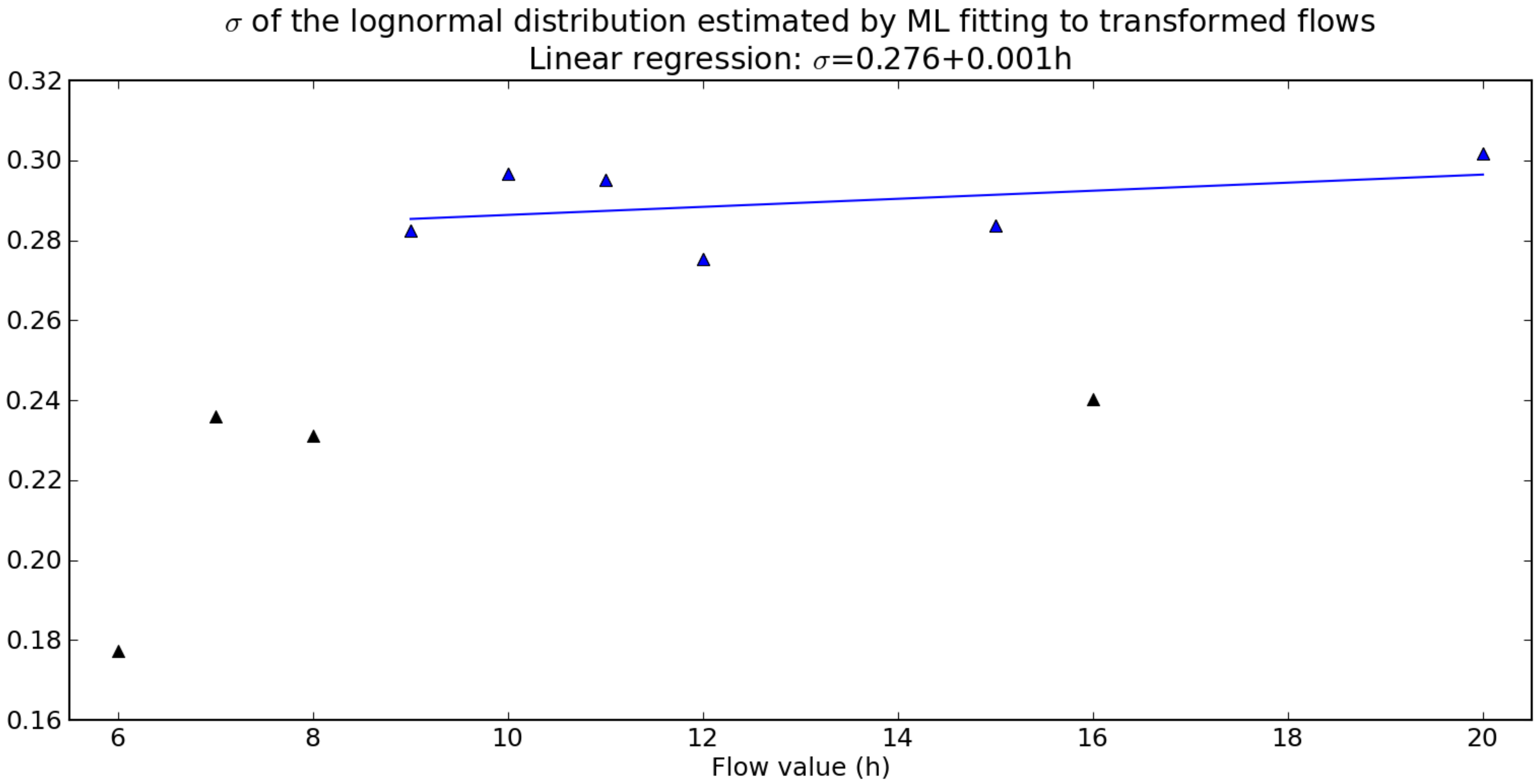
σ of the lognormal distribution estimated by ML fitting to transformed flows
Linear regression: σ=0.276+0.001h
0.32
0.30
0.28
0.26
0.24
0.22
0.20
0.18
0.16
6
8
10
12
14
16
18
20
Flow value (h)
