# Supplementary figures and images for: Evolutionary structure of Plasmodium falciparum major variant surface antigen genes in South America: Implications for epidemic transmission and surveillance
Source: Ecol Evol. 2017 Oct 8;7(22):9376–90. doi: 10.1002/ece3.3425 (PMC5696401; doi:10.1002/ece3.3425)

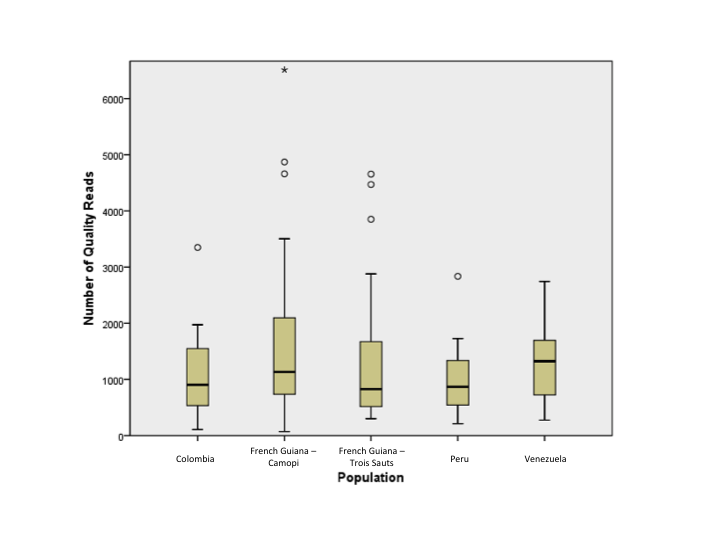

Supplement: Supplementary file 2 [file ECE3-7-9376-s002.tiff]

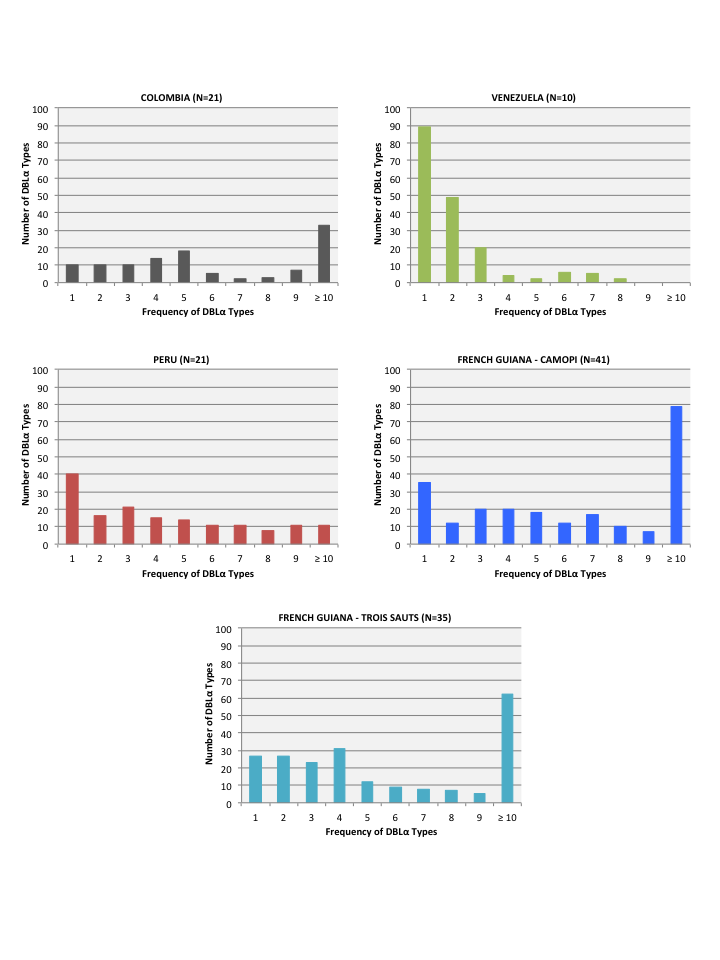

Supplement: Supplementary file 3 [file ECE3-7-9376-s003.tiff]

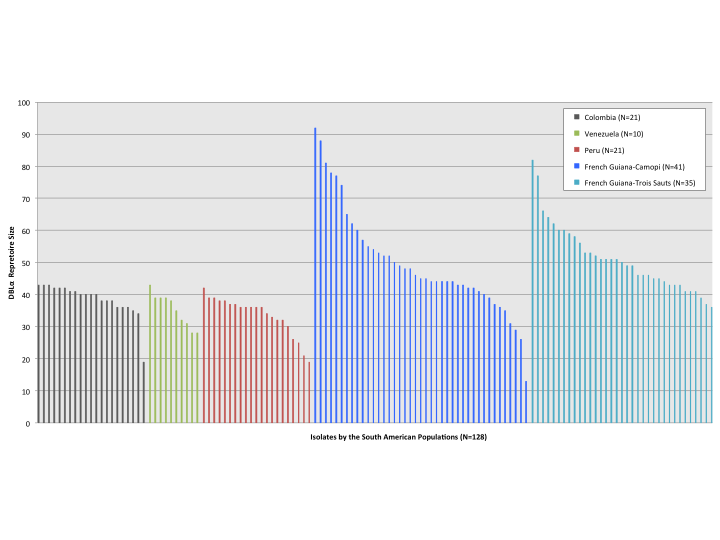

Supplement: Supplementary file 4 [file ECE3-7-9376-s004.tiff]

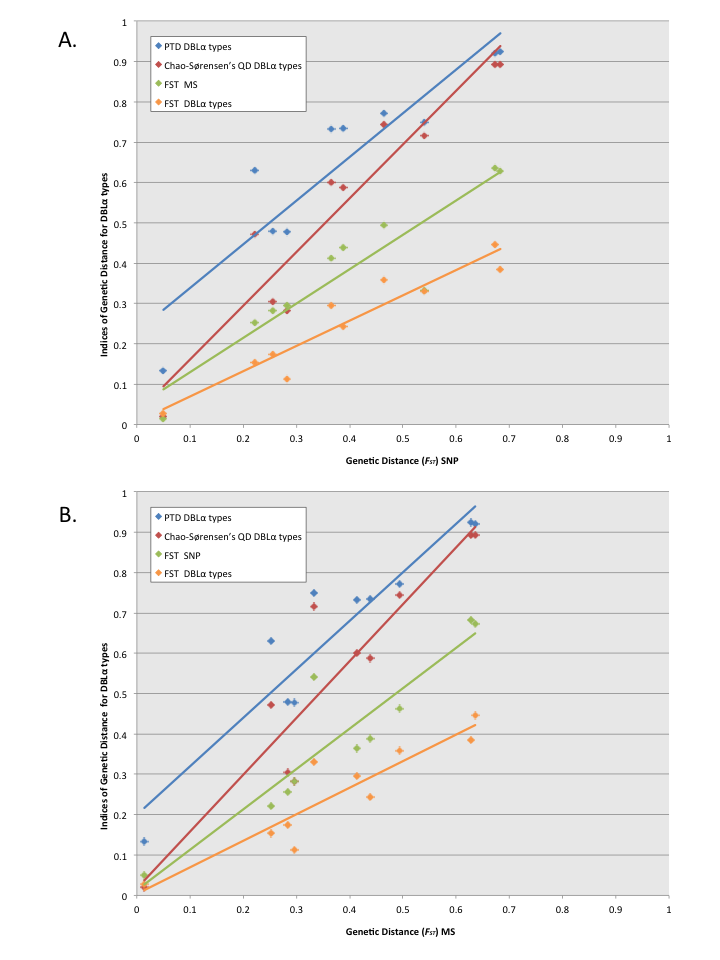

Supplement: Supplementary file 5 [file ECE3-7-9376-s005.tiff]

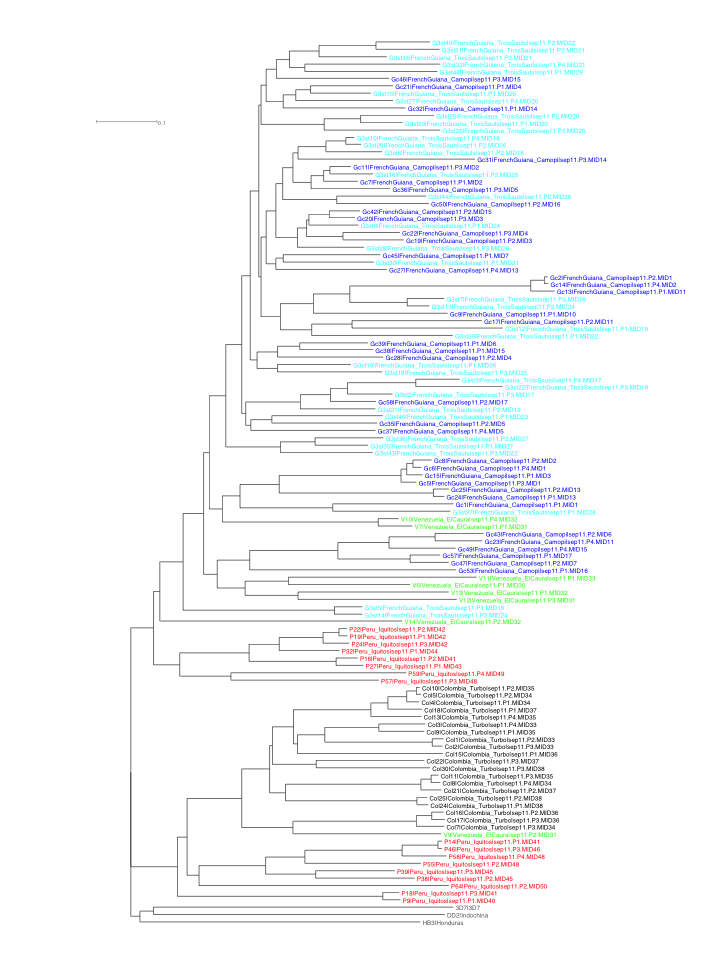

Supplement: Supplementary file 6 [file ECE3-7-9376-s006.tiff]
