# Supplementary material for: Evolutionary structure of Plasmodium falciparum major variant surface antigen genes in South America: Implications for epidemic transmission and surveillance
Source: Ecol Evol. 2017 Oct 8;7(22):9376–90. doi: 10.1002/ece3.3425 (PMC5696401; doi:10.1002/ece3.3425)
Supplement: Supplementary file 7 [file ECE3-7-9376-s007.docx]

**Supplementary Tables**

**Table S1.** List of forward fusion primer sequences used for multiplex 454 PCR amplicon sequencing. The adaptor is represented in red, the key (TCAG) in blue and the primer sequence in green. The MIDs, which are different for each primer, are in black.

| **Primer name** | **Complete Primer Sequence (5'-3')** |
| --- | --- |
| DBLaAF-MID-1 | CGTATCGCCTCCCTCGCGCCATCAGACGAGTGCGTGCACGMAGTTTYGC |
| DBLaAF-MID-2 | CGTATCGCCTCCCTCGCGCCATCAGACGCTCGACAGCACGMAGTTTYGC |
| DBLaAF-MID-3 | CGTATCGCCTCCCTCGCGCCATCAGAGACGCACTCGCACGMAGTTTYGC |
| DBLaAF-MID-4 | CGTATCGCCTCCCTCGCGCCATCAGAGCACTGTAGGCACGMAGTTTYGC |
| DBLaAF-MID-5 | CGTATCGCCTCCCTCGCGCCATCAGATCAGACACGGCACGMAGTTTYGC |
| DBLaAF-MID-6 | CGTATCGCCTCCCTCGCGCCATCAGATATCGCGAGGCACGMAGTTTYGC |
| DBLaAF-MID-7 | CGTATCGCCTCCCTCGCGCCATCAGCGTGTCTCTAGCACGMAGTTTYGC |
| DBLaAF-MID-8 | CGTATCGCCTCCCTCGCGCCATCAGCTCGCGTGTCGCACGMAGTTTYGC |
| DBLaAF-MID-10 | CGTATCGCCTCCCTCGCGCCATCAGTCTCTATGCGGCACGMAGTTTYGC |
| DBLaAF-MID-11 | CGTATCGCCTCCCTCGCGCCATCAGTGATACGTCTGCACGMAGTTTYGC |
| DBLaAF-MID-13 | CGTATCGCCTCCCTCGCGCCATCAGCATAGTAGTGGCACGMAGTTTYGC |
| DBLaAF-MID-14 | CGTATCGCCTCCCTCGCGCCATCAGCGAGAGATACGCACGMAGTTTYGC |
| DBLaAF-MID-15 | CGTATCGCCTCCCTCGCGCCATCAGATACGACGTAGCACGMAGTTTYGC |
| DBLaAF-MID-16 | CGTATCGCCTCCCTCGCGCCATCAGTCACGTACTAGCACGMAGTTTYGC |
| DBLaAF-MID-17 | CGTATCGCCTCCCTCGCGCCATCAGCGTCTAGTACGCACGMAGTTTYGC |
| DBLaAF-MID-18 | CGTATCGCCTCCCTCGCGCCATCAGTCTACGTAGCGCACGMAGTTTYGC |
| DBLaAF-MID-19 | CGTATCGCCTCCCTCGCGCCATCAGTGTACTACTCGCACGMAGTTTYGC |
| DBLaAF-MID-20 | CGTATCGCCTCCCTCGCGCCATCAGACGACTACAGGCACGMAGTTTYGC |
| DBLaAF-MID-21 | CGTATCGCCTCCCTCGCGCCATCAGCGTAGACTAGGCACGMAGTTTYGC |
| DBLaAF-MID-22 | CGTATCGCCTCCCTCGCGCCATCAGTACGAGTATGGCACGMAGTTTYGC |
| DBLaAF-MID-23 | CGTATCGCCTCCCTCGCGCCATCAGTACTCTCGTGGCACGMAGTTTYGC |
| DBLaAF-MID-24 | CGTATCGCCTCCCTCGCGCCATCAGTGAAGACGAGGCACGMAGTTTYGC |
| DBLaAF-MID-25 | CGTATCGCCTCCCTCGCGCCATCAGTCGTCGCTCGGCACGMAGTTTYGC |
| DBLaAF-MID-26 | CGTATCGCCTCCCTCGCGCCATCAGACATACGCGTGCACGMAGTTTYGC |
| DBLaAF-MID-27 | CGTATCGCCTCCCTCGCGCCATCAGACGCGAGTATGCACGMAGTTTYGC |
| DBLaAF-MID-28 | CGTATCGCCTCCCTCGCGCCATCAGACTACTATGTGCACGMAGTTTYGC |
| DBLaAF-MID-29 | CGTATCGCCTCCCTCGCGCCATCAGACTGTACAGTGCACGMAGTTTYGC |
| DBLaAF-MID-30 | CGTATCGCCTCCCTCGCGCCATCAGAGACTATACTGCACGMAGTTTYGC |
| DBLaAF-MID-31 | CGTATCGCCTCCCTCGCGCCATCAGAGCGTCGTCTGCACGMAGTTTYGC |
| DBLaAF-MID-32 | CGTATCGCCTCCCTCGCGCCATCAGAGTACGCTATGCACGMAGTTTYGC |
| DBLaAF-MID-33 | CGTATCGCCTCCCTCGCGCCATCAGATAGAGTACTGCACGMAGTTTYGC |
| DBLaAF-MID-34 | CGTATCGCCTCCCTCGCGCCATCAGCACGCTACGTGCACGMAGTTTYGC |
| DBLaAF-MID-35 | CGTATCGCCTCCCTCGCGCCATCAGCAGTAGACGTGCACGMAGTTTYGC |
| DBLaAF-MID-36 | CGTATCGCCTCCCTCGCGCCATCAGCGACGTGACTGCACGMAGTTTYGC |
| DBLaAF-MID-37 | CGTATCGCCTCCCTCGCGCCATCAGTACACACACTGCACGMAGTTTYGC |
| DBLaAF-MID-38 | CGTATCGCCTCCCTCGCGCCATCAGTACACGTGATGCACGMAGTTTYGC |
| DBLaAF-MID-39 | CGTATCGCCTCCCTCGCGCCATCAGTACAGATCGTGCACGMAGTTTYGC |
| DBLaAF-MID-40 | CGTATCGCCTCCCTCGCGCCATCAGTACGCTGTCTGCACGMAGTTTYGC |
| DBLaAF-MID-41 | CGTATCGCCTCCCTCGCGCCATCAGTAGTGTAGATGCACGMAGTTTYGC |
| DBLaAF-MID-42 | CGTATCGCCTCCCTCGCGCCATCAGTCGATCACGTGCACGMAGTTTYGC |
| DBLaAF-MID-43 | CGTATCGCCTCCCTCGCGCCATCAGTCGCACTAGTGCACGMAGTTTYGC |
| DBLaAF-MID-44 | CGTATCGCCTCCCTCGCGCCATCAGTCTAGCGACTGCACGMAGTTTYGC |
| DBLaAF-MID-45 | CGTATCGCCTCCCTCGCGCCATCAGTCTATACTATGCACGMAGTTTYGC |
| DBLaAF-MID-46 | CGTATCGCCTCCCTCGCGCCATCAGTGACGTATGTGCACGMAGTTTYGC |
| DBLaAF-MID-47 | CGTATCGCCTCCCTCGCGCCATCAGTGTGAGTAGTGCACGMAGTTTYGC |
| DBLaAF-MID-48 | CGTATCGCCTCCCTCGCGCCATCAGACAGTATATAGCACGMAGTTTYGC |
| DBLaAF-MID-49 | CGTATCGCCTCCCTCGCGCCATCAGACGCGATCGAGCACGMAGTTTYGC |
| DBLaAF-MID-50 | CGTATCGCCTCCCTCGCGCCATCAGACTAGCAGTAGCACGMAGTTTYGC |
| DBLaAF-MID-51 | CGTATCGCCTCCCTCGCGCCATCAGAGCTCACGTAGCACGMAGTTTYGC |
| DBLaAF-MID-52 | CGTATCGCCTCCCTCGCGCCATCAGAGCTCACGTAGCACGMAGTTTYGC |
| DBLaAF-MID-53 | CGTATCGCCTCCCTCGCGCCATCAGAGTCGAGAGAGCACGMAGTTTYGC |
| DBLaAF-MID-54 | CGTATCGCCTCCCTCGCGCCATCAGAGTGCTACGAGCACGMAGTTTYGC |
| DBLaAF-MID-55 | CGTATCGCCTCCCTCGCGCCATCAGCGATCGTATAGCACGMAGTTTYGC |
| DBLaAF-MID-56 | CGTATCGCCTCCCTCGCGCCATCAGCGCAGTACGAGCACGMAGTTTYGC |
| DBLaAF-MID-57 | CGTATCGCCTCCCTCGCGCCATCAGCGCGTATACAGCACGMAGTTTYGC |
| DBLaAF-MID-58 | CGTATCGCCTCCCTCGCGCCATCAGCGTACAGTCAGCACGMAGTTTYGC |
| DBLaAF-MID-59 | CGTATCGCCTCCCTCGCGCCATCAGCGTACTCAGAGCACGMAGTTTYGC |
| DBLaAF-MID-60 | CGTATCGCCTCCCTCGCGCCATCAGCTACGCTCTAGCACGMAGTTTYGC |
| DBLaAF-MID-61 | CGTATCGCCTCCCTCGCGCCATCAGCTATAGCGTAGCACGMAGTTTYGC |
| DBLaAF-MID-62 | CGTATCGCCTCCCTCGCGCCATCAGTACGTCATCAGCACGMAGTTTYGC |

**Table S2.** List of reverse fusion primer sequences used for multiplex 454 PCR amplicon sequencing. The adaptator is represented in red, the key (TCAG) in bleu and the primer sequence in green. The MID, which are different for each primer, are in black.

| **Primer name** | **Complete Primer Sequence (5'-3')** |
| --- | --- |
| DBLaBR-MID-1 | CTATGCGCCTTGCCAGCCCGCTCAGACGAGTGCGTGCCCATTCSTCGAACCA |
| DBLaBR-MID-2 | CTATGCGCCTTGCCAGCCCGCTCAGACGCTCGACAGCCCATTCSTCGAACCA |
| DBLaBR-MID-3 | CTATGCGCCTTGCCAGCCCGCTCAGAGACGCACTCGCCCATTCSTCGAACCA |
| DBLaBR-MID-4 | CTATGCGCCTTGCCAGCCCGCTCAGAGCACTGTAGGCCCATTCSTCGAACCA |
| DBLaBR-MID-5 | CTATGCGCCTTGCCAGCCCGCTCAGATCAGACACGGCCCATTCSTCGAACCA |
| DBLaBR-MID-6 | CTATGCGCCTTGCCAGCCCGCTCAGATATCGCGAGGCCCATTCSTCGAACCA |
| DBLaBR-MID-7 | CTATGCGCCTTGCCAGCCCGCTCAGCGTGTCTCTAGCCCATTCSTCGAACCA |
| DBLaBR-MID-8 | CTATGCGCCTTGCCAGCCCGCTCAGCTCGCGTGTCGCCCATTCSTCGAACCA |
| DBLaBR-MID-10 | CTATGCGCCTTGCCAGCCCGCTCAGTCTCTATGCGGCCCATTCSTCGAACCA |
| DBLaBR-MID-11 | CTATGCGCCTTGCCAGCCCGCTCAGTGATACGTCTGCCCATTCSTCGAACCA |
| DBLaBR-MID-13 | CTATGCGCCTTGCCAGCCCGCTCAGCATAGTAGTGGCCCATTCSTCGAACCA |
| DBLaBR-MID-14 | CTATGCGCCTTGCCAGCCCGCTCAGCGAGAGATACGCCCATTCSTCGAACCA |
| DBLaBR-MID-15 | CTATGCGCCTTGCCAGCCCGCTCAGATACGACGTAGCCCATTCSTCGAACCA |
| DBLaBR-MID-16 | CTATGCGCCTTGCCAGCCCGCTCAGTCACGTACTAGCCCATTCSTCGAACCA |
| DBLaBR-MID-17 | CTATGCGCCTTGCCAGCCCGCTCAGCGTCTAGTACGCCCATTCSTCGAACCA |
| DBLaBR-MID-18 | CTATGCGCCTTGCCAGCCCGCTCAGTCTACGTAGCGCCCATTCSTCGAACCA |
| DBLaBR-MID-19 | CTATGCGCCTTGCCAGCCCGCTCAGTGTACTACTCGCCCATTCSTCGAACCA |
| DBLaBR-MID-20 | CTATGCGCCTTGCCAGCCCGCTCAGACGACTACAGGCCCATTCSTCGAACCA |
| DBLaBR-MID-21 | CTATGCGCCTTGCCAGCCCGCTCAGCGTAGACTAGGCCCATTCSTCGAACCA |
| DBLaBR-MID-22 | CTATGCGCCTTGCCAGCCCGCTCAGTACGAGTATGGCCCATTCSTCGAACCA |
| DBLaBR-MID-23 | CTATGCGCCTTGCCAGCCCGCTCAGTACTCTCGTGGCCCATTCSTCGAACCA |
| DBLaBR-MID-24 | CTATGCGCCTTGCCAGCCCGCTCAGTGAAGACGAGGCCCATTCSTCGAACCA |
| DBLaBR-MID-25 | CTATGCGCCTTGCCAGCCCGCTCAGTCGTCGCTCGGCCCATTCSTCGAACCA |
| DBLaBR-MID-26 | CTATGCGCCTTGCCAGCCCGCTCAGACATACGCGTGCCCATTCSTCGAACCA |
| DBLaBR-MID-27 | CTATGCGCCTTGCCAGCCCGCTCAGACGCGAGTATGCCCATTCSTCGAACCA |
| DBLaBR-MID-28 | CTATGCGCCTTGCCAGCCCGCTCAGACTACTATGTGCCCATTCSTCGAACCA |
| DBLaBR-MID-29 | CTATGCGCCTTGCCAGCCCGCTCAGACTGTACAGTGCCCATTCSTCGAACCA |
| DBLaBR-MID-30 | CTATGCGCCTTGCCAGCCCGCTCAGAGACTATACTGCCCATTCSTCGAACCA |
| DBLaBR-MID-31 | CTATGCGCCTTGCCAGCCCGCTCAGAGCGTCGTCTGCCCATTCSTCGAACCA |
| DBLaBR-MID-32 | CTATGCGCCTTGCCAGCCCGCTCAGAGTACGCTATGCCCATTCSTCGAACCA |
| DBLaBR-MID-33 | CTATGCGCCTTGCCAGCCCGCTCAGATAGAGTACTGCCCATTCSTCGAACCA |
| DBLaBR-MID-34 | CTATGCGCCTTGCCAGCCCGCTCAGCACGCTACGTGCCCATTCSTCGAACCA |
| DBLaBR-MID-35 | CTATGCGCCTTGCCAGCCCGCTCAGCAGTAGACGTGCCCATTCSTCGAACCA |
| DBLaBR-MID-36 | CTATGCGCCTTGCCAGCCCGCTCAGCGACGTGACTGCCCATTCSTCGAACCA |
| DBLaBR-MID-37 | CTATGCGCCTTGCCAGCCCGCTCAGTACACACACTGCCCATTCSTCGAACCA |
| DBLaBR-MID-38 | CTATGCGCCTTGCCAGCCCGCTCAGTACACGTGATGCCCATTCSTCGAACCA |
| DBLaBR-MID-39 | CTATGCGCCTTGCCAGCCCGCTCAGTACAGATCGTGCCCATTCSTCGAACCA |
| DBLaBR-MID-40 | CTATGCGCCTTGCCAGCCCGCTCAGTACGCTGTCTGCCCATTCSTCGAACCA |
| DBLaBR-MID-41 | CTATGCGCCTTGCCAGCCCGCTCAGTAGTGTAGATGCCCATTCSTCGAACCA |
| DBLaBR-MID-42 | CTATGCGCCTTGCCAGCCCGCTCAGTCGATCACGTGCCCATTCSTCGAACCA |
| DBLaBR-MID-43 | CTATGCGCCTTGCCAGCCCGCTCAGTCGCACTAGTGCCCATTCSTCGAACCA |
| DBLaBR-MID-44 | CTATGCGCCTTGCCAGCCCGCTCAGTCTAGCGACTGCCCATTCSTCGAACCA |
| DBLaBR-MID-45 | CTATGCGCCTTGCCAGCCCGCTCAGTCTATACTATGCCCATTCSTCGAACCA |
| DBLaBR-MID-46 | CTATGCGCCTTGCCAGCCCGCTCAGTGACGTATGTGCCCATTCSTCGAACCA |
| DBLaBR-MID-47 | CTATGCGCCTTGCCAGCCCGCTCAGTGTGAGTAGTGCCCATTCSTCGAACCA |
| DBLaBR-MID-48 | CTATGCGCCTTGCCAGCCCGCTCAGACAGTATATAGCCCATTCSTCGAACCA |
| DBLaBR-MID-49 | CTATGCGCCTTGCCAGCCCGCTCAGACGCGATCGAGCCCATTCSTCGAACCA |
| DBLaBR-MID-50 | CTATGCGCCTTGCCAGCCCGCTCAGACTAGCAGTAGCCCATTCSTCGAACCA |
| DBLaBR-MID-51 | CTATGCGCCTTGCCAGCCCGCTCAGAGCTCACGTAGCCCATTCSTCGAACCA |
| DBLaBR-MID-52 | CTATGCGCCTTGCCAGCCCGCTCAGAGTATACATAGCCCATTCSTCGAACCA |
| DBLaBR-MID-53 | CTATGCGCCTTGCCAGCCCGCTCAGAGTCGAGAGAGCCCATTCSTCGAACCA |
| DBLaBR-MID-54 | CTATGCGCCTTGCCAGCCCGCTCAGAGTGCTACGAGCCCATTCSTCGAACCA |
| DBLaBR-MID-55 | CTATGCGCCTTGCCAGCCCGCTCAGCGATCGTATAGCCCATTCSTCGAACCA |
| DBLaBR-MID-56 | CTATGCGCCTTGCCAGCCCGCTCAGCGCAGTACGAGCCCATTCSTCGAACCA |
| DBLaBR-MID-57 | CTATGCGCCTTGCCAGCCCGCTCAGCGCGTATACAGCCCATTCSTCGAACCA |
| DBLaBR-MID-58 | CTATGCGCCTTGCCAGCCCGCTCAGCGTACAGTCAGCCCATTCSTCGAACCA |
| DBLaBR-MID-59 | CTATGCGCCTTGCCAGCCCGCTCAGCGTACTCAGAGCCCATTCSTCGAACCA |
| DBLaBR-MID-60 | CTATGCGCCTTGCCAGCCCGCTCAGCTACGCTCTAGCCCATTCSTCGAACCA |
| DBLaBR-MID-61 | CTATGCGCCTTGCCAGCCCGCTCAGCTATAGCGTAGCCCATTCSTCGAACCA |
| DBLaBR-MID-62 | CTATGCGCCTTGCCAGCCCGCTCAGTACGTCATCAGCCCATTCSTCGAACCA |

**Supplementary Figure Legends**

**Figure S1. Flow value distributions for different homopolymer lengths in the control samples 3D7, Dd2, and HB3.** Distributions suggested in Balzer et al. are shown together with fitted and extrapolated log-normal distributions (Balzer et. 2010). Linear extrapolation of maximum likelihood estimated parameters μ and σ is shown in the last two panels.

**Figure S2.** **Boxplot of the distribution of the number of quality reads obtained per isolate, grouped by South American population.** The box represents the interquartile range, the whiskers represent all values up to 1.5x the interquartile range, and the circles and stars are outliers, i.e. beyond 1.5x the interquartile range.

**Figure S3.** **Frequency distribution of *var* DBLα types in each South American population.** The x-axis is the frequency class for each ***var*** DBLα type, arranged from lowest (observed once) to the highest (observed ≥10). The y-axis depicts the number of unique ***var*** DBLα types found in each frequency class. The frequency distributions have been subdivided for each South American population. In parentheses next to each population is the number (N) of isolates sampled from the population.

**Figure S4. The distribution of *var* DBLα repertoire sizes the 128 isolates surveyed in the South American population.**

**Figure S5.** **Estimated correlation of genetic differentiation.** (A) Correlation between the pairs of populations for genetic distance using *F_ST_* for SNPs plotted against the indices of genetic distance for each data set: PTD ***var*** DBLα s, Chao-Sørensen’s QD ***var*** DBLα s, *F_ST_* MS, and *F_ST_* ***var*** DBLα s. The Pearson’s correlation coefficients (*r*) were calculated between the various indices of genetic distance and were determined to be as follows: PTD ***var*** DBLα s (*r* = 0.92, blue), Chao-Sørensen’s QD ***var*** DBLα s (*r* = 0.95, red), *F_ST_* MS (*r* = 0.92, green), and *F_ST_* ***var*** DBLα s (*r* = 0.95, orange). (B) Correlation between the pairs of populations for genetic distance using *F_ST_* for MS plotted against the indices of genetic distance for each data set: PTD ***var*** DBLα s, Chao-Sørensen’s QD ***var*** DBLα s, *F_ST_* SNPs, and *F_ST_* ***var*** DBLα s. The Pearson’s correlation coefficients (*r*) were calculated between the various indices of genetic distance and were determined to be as follows: PTD ***var*** DBLα s (*r* = 0.94, blue), Chao-Sørensen’s QD ***var*** DBLα s (*r* = 0.93, red), *F_ST_* SNP (*r* = 0.92, green), and ***var*** *F_ST_* DBLα s (*r* = 0.92, orange).

**Figure S6.** **Neighbor joining tree based on the isolate distance measure using PTD and with the out-group of 3D7, Dd2 and HB3.** The Venezuelan samples are represented in green those of Colombia in grey, of Peru in red, of French Guiana-Camopi in blue and of French Guiana-Trois Sauts in turquoise (Note: to allow for DBLα comparisons isolates with < 30 sequences were excluded for this analysis).
